# Gut Microbiota Enterotypes Mediate the Effects of Dietary Patterns on Colorectal Neoplasm Risk in a Chinese Population

**DOI:** 10.3390/nu15132940

**Published:** 2023-06-28

**Authors:** Jia-An Cai, Yong-Zhen Zhang, En-Da Yu, Wei-Qun Ding, Qing-Wu Jiang, Quan-Cai Cai, Liang Zhong

**Affiliations:** 1 Department of Gastroenterology and Endoscopy, Huashan Hospital, Fudan University, Shanghai 200040, China; 16301050206@fudan.edu.cn (J.-A.C.); dingweiqun@huashan.org.cn (W.-Q.D.); 2 Department of Gastroenterology, Changhai Hospital, Naval Medical University, Shanghai 200433, China; zyzzhangyongzhen@163.com; 3 Department of Gastroenterology, 928 Hospital of PLA Joint Logistics Force, Haikou 570100, China; 4 Department of General Surgery, Changhai Hospital, Naval Medical University, Shanghai 200433, China; endayu03@163.com; 5 Key Laboratory of Public Health Safety of Ministry of Education, School of Public Health, Fudan University, Shanghai 200032, China; 6 National Clinical Research Center for Digestive Diseases, Shanghai 200433, China

**Keywords:** dietary patterns, gut microbiota, enterotypes, colorectal cancer, colorectal adenoma

## Abstract

Colorectal cancer (CRC) risk is influenced by dietary patterns and gut microbiota enterotypes. However, the interaction between these factors remains unclear. This study examines this relationship, hypothesizing that different diets may affect colorectal tumor risk in individuals with varied gut microbiota enterotypes. We conducted a case-control study involving 410 Han Chinese individuals, using exploratory structural equation modeling to identify two dietary patterns, and a Dirichlet multinomial mixture model to classify 250 colorectal neoplasm cases into three gut microbiota enterotypes. We assessed the association between dietary patterns and the risk of each tumor subtype using logistic regression analysis. We found that a healthy diet, rich in vegetables, fruits, milk, and yogurt, lowers CRC risk, particularly in individuals with type I (dominated by *Bacteroides* and *Lachnoclostridium*) and type II (dominated by *Bacteroides* and *Faecalibacterium*) gut microbiota enterotypes, with adjusted odds ratios (ORs) of 0.66 (95% confidence interval [CI] = 0.48–0.89) and 0.42 (95% CI = 0.29–0.62), respectively. Fruit consumption was the main contributor to this protective effect. No association was found between a healthy dietary pattern and colorectal adenoma risk or between a high-fat diet and colorectal neoplasm risk. Different CRC subtypes associated with gut microbiota enterotypes displayed unique microbial compositions and functions. Our study suggests that specific gut microbiota enterotypes can modulate the effects of diet on CRC risk, offering new perspectives on the relationship between diet, gut microbiota, and colorectal neoplasm risk.

## 1. Introduction

Colorectal cancer (CRC) is one of the most common and deadly malignancies worldwide, accounting for approximately 10% of all cancer cases and fatalities [1]. CRC development is influenced by both genetic and environmental factors, among which diet plays a pivotal role. Several dietary patterns have been associated with different CRC risks, such as the Western diet (characterized by high intakes of red meat, processed meat, refined grains, and sugar-sweetened beverages) [2], the prudent diet (marked by high intakes of fruits, vegetables, whole grains, fish, and poultry) [3], the Mediterranean diet (emphasizing high intakes of olive oil, legumes, nuts, fruits, vegetables, fish, and moderate wine consumption) [4], and the Asian diet (featuring high intakes of rice, soy products, vegetables, fish, and seaweed) [5]. However, the mechanisms by which dietary patterns modulate CRC risk remain poorly understood.

One potential explanation is that dietary patterns influence the composition and function of gut microbiota—a complex ecosystem of microorganisms inhabiting the human gastrointestinal tract [6]. Gut microbiota is involved in various aspects of human health and disease, particularly CRC pathogenesis. Previous studies have shown that CRC patients have altered gut microbiota compared to healthy controls, indicating a dysbiosis between beneficial and harmful bacteria [7]. Dietary patterns can modulate the composition and function of gut microbiota, which in turn can affect human health and disease [8]. Different dietary components, such as carbohydrates, proteins, fats, fibers, polyphenols, and vitamins, can affect the abundance and diversity of gut microbes, as well as their metabolic activities and interactions [6]. Additionally, dietary patterns can also influence the functional potential of gut microbiota, as revealed by metagenomic and metabolomic analyses [8,9]. For example, a Western dietary pattern has been associated with lower levels of genes related to short-chain fatty acids (SCFAs) synthesis, amino acid metabolism, and bile acid transformation in the gut microbiome [6,10]. Moreover, different gut microbiota enterotypes have been identified based on their predominant bacterial genera, such as *Bacteroides* (enterotype 1), *Prevotella* (enterotype 2), and *Ruminococcus* (enterotype 3) [11]. These enterotypes may reflect different metabolic capacities and responses to dietary interventions.

Based on these findings, we hypothesized that different dietary patterns could affect the risk of developing colorectal tumors, including CRC and colorectal adenoma (CRA), in individuals with different gut microbiota enterotypes. To test this hypothesis, we conducted a case-case-control study to identify dietary patterns and gut microbiota enterotypes, and to evaluate their association with colorectal tumor risk. We also compared the diversity, composition, and function of gut microbiota among different tumor subtypes to better understand how dietary patterns influence tumor risk by modulating gut microbiota. Our study aims to provide valuable insights into the relationship between dietary patterns, gut microbiota, and colorectal tumor risk, potentially informing future preventive strategies.

## 2. Materials and Methods

### 2.1. Study Population

We conducted a case-control study involving 410 Han Chinese individuals aged 40 or older who underwent colonoscopy at Changhai Hospital (Shanghai, China) between 2015 and 2016. We applied exclusion criteria to ensure the validity of our results. The inclusion and exclusion criteria are summarized in Table S1 in the Supplementary Materials. The CRC group and CRA group comprised patients diagnosed with CRC or CRA, respectively, after colonoscopy. The control group consisted of individuals diagnosed with hyperplastic polyps or those with no significant findings after colonoscopy. We classified participants with multiple tumors according to the most advanced pathological changes. We defined the proximal colon as the caecum, ascending colon, hepatic flexure, transverse colon, and splenic flexure, and the distal colon as the descending colon, sigmoid colon, and rectosigmoid junction.

In this study, we considered any malignant or premalignant lesions in the colorectum, including CRC and CRA, as colorectal neoplasms. We diagnosed CRC by histopathological confirmation of invasive adenocarcinoma in the colorectum and CRA by histopathological confirmation of tubular adenoma, tubulovillous adenoma, or villous adenoma in the colorectum. We also classified colorectal neoplasms into three subtypes based on their gut microbiota profiles using the Dirichlet multinomial mixture model (DMM) (see Section 2.5.3 and Figure S1 in Section 3 for details).

We recruited participants from three sources at Changhai Hospital: the gastroenterology clinic, the general surgery clinic, and the health examination center. Research staff screened potential participants based on a brief medical history interview to determine their eligibility for the study.

### 2.2. Study Procedures

We invited eligible participants to join the study on site and obtained their informed consent. They then completed a self-reported questionnaire on potential risk factors, such as age, sex, body mass index (BMI), education degree, physical activity, smoking, drinking, dietary intake, and other factors, as previously described [12]. We used a simple semiquantitative food frequency questionnaire to assess dietary intake, including green vegetables, fresh fruits, milk, yoghurt, pickled food, fried food, red meat (beef, pork, and lamb), and white meat (fish, chicken, and duck). Participants reported the average frequency of each food item consumed during the past year as occasional (less than 3 times per week) or regular (at least 3 times per week).

We collected fresh stool samples (≥1 g) from participants and stored them in a −80 °C refrigerator for subsequent DNA extraction and metabolomics analysis. We performed DNA extraction using the OMEGA-soil DNA Isolation Kit (USA Omega Bio-Tek, Norcross, GA, USA) and 16S rDNA sequencing on the Illumina MiSeq platform (Illumina, San Diego, CA, USA) [13]. We also performed metabolomics analysis using Agilent 1290 Infinity UHPLC and Agilent 6538 UHD and Accurate-Mass Q-TOF/MS according to a previously reported protocol [14]. We describe the details of these analytical methods in Section 2.3 and Section 2.4, respectively.

Participants underwent colonoscopy within two or three days of completing the questionnaire and providing stool samples. The colonoscopy procedure was consistent with our previous study [12].

### 2.3. 16S rDNA Sequencing and Data Processing

We amplified the V3-V4 region of the bacterial 16S rDNA gene using the primers 341F (5′-CCTACGGGNGGCWGCAG-3′) and 805R (5′-GACTACHVGGGTATCTAATCC-3′). We purified the PCR products using the AxyPrep DNA Gel Extraction Kit (Axygen Biosciences, Union City, CA, USA) and quantified them using QuantiFluor-ST (Promega, Madison, WI, USA). We pooled the purified amplicons in equimolar amounts and performed paired-end sequencing (2 × 300) on an Illumina MiSeq platform (Illumina, San Diego, CA, USA) according to the standard protocols.

We quality filtered the raw reads using Trimmomatic v0.27 (Usadel Lab, German Center for Biotechnology, Aachen, Germany) with the following parameters: LEADING:3 TRAILING:3 SLIDINGWINDOW:4:15 MINLEN:200. We merged the paired-end reads using FLASH v1.2.11 (The FLASH Team, University of Illinois at Urbana-Champaign, Champaign, IL, USA) with a minimum overlap of 10 bp and a maximum mismatch density of 0.25 [15]. We identified and removed the chimeric sequences using Usearch v7.1 (Robert C. Edgar, Drive5 Bioinformatics, Mill Valley, CA, USA) with the reference database Gold.fa. We clustered the remaining high-quality sequences into operational taxonomic units (OTUs) at 97% similarity using Usearch v7.1 with the UPARSE algorithm. We assigned the representative sequence of each OTU to a taxonomic level using QIIME v1.9.1 (The QIIME Development Group, multiple institutions and laboratories in the USA, Canada, Europe and Australia) with the RDP Classifier v2.2 (Ribosomal Database Project, Michigan State University, East Lansing, MI, USA) and the Silva database (Release 123) (SILVA Team, Max Planck Institute for Computer Science and the Institute for Microbial Ecology at the University of Bremen, Germany) [16,17].

We obtained the taxonomic information corresponding to each OTU by counting the community composition of each sample at each taxonomic level (domain, phylum, class, order, family, genus).

### 2.4. Metabolome Analysis

We thawed fecal samples at room temperature, suspended them in a methanol: water (8:2) solvent, and obtained the supernatant by centrifugation. We used a Waters XSelect HSS T3 chromatographic column on the UHPLC-Q-TOFMS platform and performed mass spectrometry analysis in both positive and negative ion modes. We executed data preprocessing, including peak detection, retention time correction, and integration, using XCMS on the R software platform (version 4.1.2) [18]. We conducted data acquisition using Agilent Masshunter Qualitative Analysis B.04.00 software, generating total ion current chromatograms for fecal samples in both ionization modes. We identified a total of 1755 features in the positive ion mode and 606 features in the negative ion mode. We imported the processed data into Simca-P software (version 11.0), where we performed centering and Pareto scaling before multivariate statistical analysis. We included quality control samples in the analysis to ensure system stability.

### 2.5. Statistical Analysis

We describe the statistical analyses we performed to address our research questions in this section. Table S2 summarizes the statistical parameters and methods we used for each analysis. We conducted all statistical analyses using IBM SPSS Statistics for Windows (version 26.0), R for Windows (version 4.1.2), and Mplus (version 8.3), with a two-tailed *p* value of less than 0.05 considered statistically significant.

#### 2.5.1. Identification of Dietary Patterns

To identify dietary patterns based on the consumption frequency of eight food items, we used exploratory structural equation modeling (ESEM), a statistical technique that allows for cross-loadings between factors and indicators. ESEM provides a more realistic representation of dietary patterns than traditional methods such as principal component analysis (PCA) or factor analysis (FA) [19]. We extracted dietary patterns using oblique rotation and determined the number of factors based on eigenvalues greater than 1.0 and the interpretability of the factors. We named each dietary pattern according to the food item with high factor loadings (≥0.3 or ≤−0.3). We calculated factor scores for each participant by summing the products of factor loadings and standardized intakes of each food item within a pattern. Higher factor scores indicated greater adherence to a specific dietary pattern.

#### 2.5.2. Relationship between Dietary Patterns and Risk of Colorectal Neoplasm

We conducted univariate analyses to compare each case group (CRC and CRA) with the control group and assess the associations of potential risk factors with colorectal neoplasms. We used the chi-square test for categorical variables and the unpaired *t*-test or Mann-Whitney U test for continuous variables. We then used binary logistic backward stepwise regression analyses to investigate the association between dietary patterns and colorectal neoplasms, controlling for other potential risk factors that had a *p* value of <0.10 in the univariate analyses.

#### 2.5.3. Relationship between Dietary Patterns and Risk of Colorectal Neoplasm Subtypes

We applied DMM using the R package “DirichletMultinomial” and clustered all 250 cases, including CRC and CRA cases, into gut microbiota enterotypes based on their OTU abundance profiles. DMM is a probabilistic method for community typing of microbial data that can infer the optimal number of community types [20]. We assigned each case to the most probable enterotype based on its posterior probability.

We used similar univariate and multivariate analyses to evaluate the associations between dietary patterns and the risk of colorectal neoplasm subtypes in our two case-case-control studies, as described previously. We conducted a Wald test to assess heterogeneity between different subtypes of colorectal tumors in relation to dietary patterns [21], aiming to determine whether there was a significant difference in their associations. In the case of significant heterogeneity, we compared the differences in gut microbiota diversity, composition, and function between different tumor subtypes to gain a better understanding of how dietary patterns influence tumor risk.

#### 2.5.4. Gut Microbiota Composition Analysis between Subgroups

We used the Kruskal-Wallis test to compare alpha-diversity among CRC or CRA subgroups, including microbial abundance indices (Chao and abundance-based coverage estimator [ACE]) and diversity indices (Shannon and Simpson). If the Kruskal-Wallis test indicated significant differences, we performed post-hoc pairwise comparisons with Dunn’s test for multiple comparisons. We visualized beta-diversity between subgroups using principal coordinates analysis (PCoA) based on Bray-Curtis distances. Moreover, we performed permutational multivariate analysis of variance (PERMANOVA) with distance matrices to ascertain significant differences in microbial communities while accounting for potential confounding factors.

We used the linear discriminant analysis (LDA) effect size (LEfSe) method to investigate alterations in gut microbiota composition among subtypes [22]. The significance criteria for identifying differentially abundant features were (1) a Kruskal-Wallis test *p* value < 0.05 and (2) a logarithmic LDA score > 3. We visualized the results of the LEfSe analysis using LDA score plots and cladograms to effectively convey the observed differences in microbial composition between subtypes.

#### 2.5.5. Metabolomics-Based Analysis of Gut Microbiota Functional Differences between Subgroups

To evaluate the functional disparities in gut microbiota among subtypes within both CRC and CRA groups, we performed metabolomic profiling, which included several specific analyses. First, we preprocessed the metabolite peak area data using logarithmic transformation and Z-score standardization. Then, we used PCoA based on Manhattan distances to assess the overall metabolite feature differences between subtypes in the CRC and CRA groups. Next, we applied the LEfSe method to identify significantly distinct metabolites between these subtypes, using a Kruskal-Wallis test *p* value < 0.05 and a logarithmic LDA score > 2 as the significance criteria. To investigate the metabolic pathway variations between the subtypes, we mapped the selected differential metabolites to well-established metabolic pathways using the Kyoto Encyclopedia of Genes and Genomes (KEGG) [23]. Moreover, we conducted pathway enrichment and pathway topology analyses using MetaboAnalyst 5.0 [24]. Finally, we assessed the correlations between differential metabolites and differential bacterial genera in subtypes using Spearman’s correlation coefficient, controlling the false discovery rate (FDR) with the FDR correction.

## 3. Results

We included three groups in this study: the CRC group with 130 patients, the CRA group with 120 patients, and a control group consisting of 160 participants. Table 1 presents the characteristics of all participants. Using ESEM, we identified two distinct dietary patterns from eight food items, which we termed the healthy dietary pattern and the high-fat dietary pattern (Table S3). The healthy dietary pattern is characterized by high consumption of vegetables, fruits, milk, and yogurt, and low intake of high-fat food products. Conversely, the high-fat dietary pattern is marked by high consumption of pickled, fried, and red meat products, and low intake of healthy foods.

Participants with higher healthy dietary scores were predominantly female, older, more educated, and had lower smoking and drinking rates (Table S4). On the other hand, those with higher high-fat dietary scores were more likely to be male, younger, less educated, have higher BMIs, and higher smoking and drinking rates (Table S4).

We explored the associations between dietary patterns and the overall risk of colorectal neoplasms. After adjusting for potential confounders, we found that higher adherence to a healthy dietary pattern was associated with a lower risk of CRC (adjusted odds ratio [OR] = 0.62, 95% confidence interval [CI] = 0.48–0.81, *p* = 0.001) (Table 2). The adjusted OR for overall CRC was 0.38 (95% CI = 0.20–0.71; *p* = 0.001 for trend) for participants in the highest tertile of healthy dietary scores compared to those in the lowest tertile (Table 3). However, we found no associations between a healthy dietary pattern and the overall risk of CRA or between a high-fat dietary pattern and the overall risk of colorectal neoplasms (Table 2 and Table 3).

We applied DMM to analyze 250 colorectal neoplasm cases and identified three unique microbial community profiles (enterotypes or subtypes), designated as type I, type II, and type III (Figure S1). Figure S2 shows that each enterotype has distinct microbial compositions, with type I dominated by *Bacteroides*, *Lachnoclostridium*, and *Escherichia shigella*; type II dominated by *Bacteroides*, *Faecalibacterium*, and *Phascolarctobacterium*; and type III characterized by *Prevotella 9*, *Bacteroides*, and *Faecalibacterium*.

We further examined the relationship between dietary patterns and colorectal neoplasm risk, stratified by gut microbiota enterotypes. Our results revealed that higher adherence to a healthy dietary pattern was associated with a reduced risk of type I CRC (adjusted OR = 0.66, 95% CI = 0.48–0.89, *p* = 0.006) and type II CRC (adjusted OR = 0.42, 95% CI = 0.29–0.62, *p* < 0.001), compared to lower adherence (Table 2). Participants in the highest tertile of healthy dietary scores showed a trend of negative associations with type I CRC (adjusted OR = 0.52, 95% CI = 0.25–1.11; *p* = 0.054 for trend) and a strong negative association with type II CRC (adjusted OR = 0.19, 95% CI = 0.07–0.48; *p* < 0.001 for trend) (Table 3). Although we observed a trend of negative associations between a healthy dietary pattern and the risk of type I CRA and type II CRA, this trend did not reach statistical significance. We found no association between a healthy dietary pattern and the risk of type III colorectal neoplasm, nor did we detect any association between a high-fat dietary pattern and the risk of colorectal neoplasm subtypes (Table 2 and Table 3). The association between a healthy dietary pattern and colorectal neoplasm risk differed significantly by gut microbiota enterotypes (type I or type II vs. type III: *p* < 0.05 for heterogeneity; type I vs. type II: *p* > 0.05 for heterogeneity).

To investigate the potential role of specific food items in explaining the differential associations between a healthy dietary pattern and the risk of colorectal neoplasm, both overall and subclassified by gut microbiota enterotypes, we analyzed the top four contributing food items to the healthy dietary pattern: vegetables, fruits, milk, and yogurt (Table S5). Our findings revealed that only fruit exhibited a similar pattern to the healthy dietary pattern, with a significant reduction in the risk of both overall colorectal neoplasm and its subtypes.

Furthermore, we investigated the relationship between the healthy dietary pattern and the risk of colorectal neoplasms, classified by lesion site, both overall and by subtype (Table S6). Our analysis revealed that a higher score for the healthy dietary pattern was associated with a lower overall risk of colorectal neoplasms in both the proximal and distal colon and rectum. In the distal colon and rectum, participants in the highest tertile of the healthy dietary pattern score had significantly negative associations with type I and type II colorectal neoplasms compared to those in the lowest tertile (all *p* < 0.05 for trend). The association between the healthy dietary pattern score and colorectal neoplasm risk varied significantly by gut microbiota enterotypes (type I or type II vs. type III: all *p* = 0.002 for heterogeneity; type I vs. type II: *p* = 0.826 for heterogeneity). However, no significant heterogeneity was observed between the subgroups in the proximal colon.

In both CRC and CRA groups, the median values of Chao, ACE, Shannon, and Simpson indices demonstrated subtype-dependent trends, with significant differences observed among subtypes (Kruskal-Wallis tests, all *p* < 0.001). Post-hoc Dunn’s test identified both significant and non-significant pairwise comparisons within groups (Table S7). In the PCoA plots (Figure S3), distinct microbial community profiles were evident in colorectal neoplasm, CRC, and CRA samples. Type I and type III groups showed minimal overlap, indicating unique microbial community structures, whereas type II overlapped with both type I and type III, suggesting a more heterogeneous microbial composition. PERMANOVA analysis further supported these findings (R^2^ = 0.08–0.12, all *p* = 0.001). The results highlight the diverse microbial environments in colorectal tumors and may help to understand the heterogeneity in associations between the healthy dietary pattern and tumor subtypes. Further investigation will focus on comparing the microbial compositions and functions between type I and type III.

**Figure 1:**
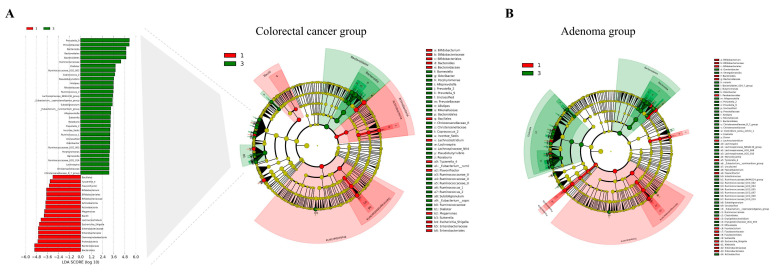
Gut microbiota composition comparison between type I and type III subtypes in CRC and CRA groups using LEfSe analysis. (**A**) LEfSe analysis of gut microbiota differences in the CRC group. Left panel: LDA score plot, with red bars representing type I taxa and green bars representing type III taxa. Right panel: cladogram displaying the phylogenetic distribution of differentially abundant taxa in the CRC group. (**B**) Cladogram illustrating the phylogenetic distribution of differentially abundant taxa between type I and type III subtypes in the CRA group, as analyzed by the LEfSe method. CRA, colorectal adenoma; CRC, colorectal cancer; LDA, linear discriminant analysis; LEfSe, linear discriminant analysis effect size.

In our study, we utilized the LEfSe analysis method to investigate the gut microbiota composition and identify differentially abundant taxa between type I and type III subtypes in both CRC and CRA groups (Figure 1). The results showed 44 bacterial genera with significant differences in abundance when comparing type I and type III subtypes across both groups (Table S8). Taxa that were more abundant in both type I CRC and type I CRA included multiple taxa from the *Proteobacteria* phylum, *Gammaproteobacteria* class, *Enterobacteriales* order, *Enterobacteriaceae* family, and *Escherichia shigella* genus. Additionally, taxa from the *Bifidobacteriales* order, *Bifidobacteriaceae* family, and *Bifidobacterium* genus exhibited higher abundance in type I subtypes. Other genera, such as *Bacteroides*, *Flavonifractor*, *Tyzzerella 4*, and *Lachnoclostridium*, were also more abundant in type I subtypes. In contrast, taxa more prevalent in type III colorectal neoplasms subtypes belonged to the *Bacteroidetes* phylum and *Bacteroidia* class. These included genera such as *Prevotella 9*, *Alistipes*, *Alloprevotella*, *Prevotella 2*, and *Odoribacter*.

**Figure 2:**
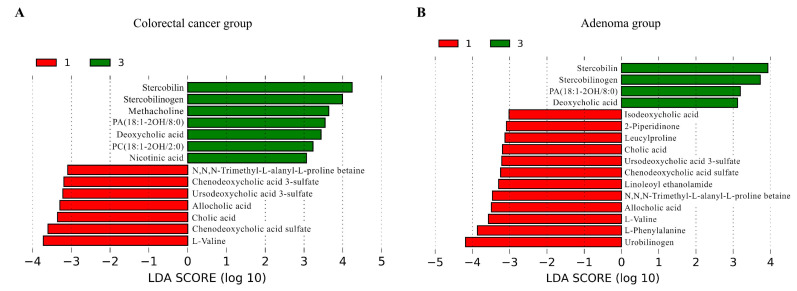
Differential metabolites between type I and type III subtypes in CRC and CRA groups identified by LEfSe analysis, with a logarithmic LDA score > 3. (**A**) LDA score plot for the CRC group comparison. (**B**) LDA score plot for the CRA group comparison. Red bars on the left represent type I, while green bars on the right indicate type III. Longer bars signify a greater degree of difference between the subtypes for each metabolite. CRA, colorectal adenoma; CRC, colorectal cancer; LDA, linear discriminant analysis; LEfSe, linear discriminant analysis effect size.

Metabolomic profiling using PCoA revealed significant differences in overall metabolite profiles between type I and type III subtypes in both CRC and CRA groups (Figure S4). Subsequently, we employed the LEfSe method to identify differential metabolites between the subtypes (Figure 2, Tables S9 and S10), which revealed 50 metabolites with significant differences in both comparisons (Table S11). The type I subtypes in both groups were characterized by elevated levels of l-valine, chenodeoxycholic acid sulfate, cholic acid, allocholic acid, ursodeoxycholic acid 3-sulfate, and *N*,*N*,*N*-trimethyl-l-alanyl-l-proline betaine, while the type III subtypes in both groups showed increased levels of stercobilin, stercobilinogen, PA(18:1–2OH/8:0), and deoxycholic acid.

We conducted a pathway analysis on 96 differentially expressed metabolites between type I and type III CRC groups (Figure S5A, Table S12). The results showed that the fatty acid degradation, tryptophan metabolism, and primary bile acid biosynthesis pathways were significantly enriched in type I CRC (all FDR adjusted *p* < 0.05), with their corresponding matched differential metabolites being l-palmitoylcarnitine, tryptamine, and cholic acid. The purine metabolism pathway was significantly enriched in type III CRC (FDR adjusted *p* < 0.05), with its corresponding matched differential metabolites being adenosine, hypoxanthine, and inosine. Additionally, thiamine metabolism, sphingolipid metabolism, and nicotinate and nicotinamide metabolism pathways were significantly enriched in type III CRC (all FDR adjusted *p* < 0.05), with their corresponding matched differential metabolites being thiamine, sphinganine, and nicotinic acid. Furthermore, the differential metabolites that matched with the pathways in sphingolipid metabolism, tryptophan metabolism, purine metabolism, and glycerophospholipid metabolism had a significant contribution to these pathways with impact values greater than zero.

We performed a pathway analysis on 111 differentially expressed metabolites between type I and type III CRA groups (Figure S5B, Table S13). The results showed that the pathways of tryptophan metabolism, tyrosine metabolism, fatty acid degradation, one-carbon pool by folate, primary bile acid biosynthesis, and glycerophospholipid metabolism were significantly enriched in type I CRA (all FDR adjusted *p* < 0.05), with their corresponding matched differential metabolites being tryptamine, tyramine, l-palmitoylcarnitine, 5-methyltetrahydrofolic acid, cholic acid, and LysoPC(16:0/0:0). The pathway of aminoacyl-tRNA biosynthesis was also significantly enriched in type I CRA (FDR adjusted *p* < 0.05), with its corresponding matched differential metabolites being l-phenylalanine and l-valine. The pathways of valine, leucine, and isoleucine degradation; valine, leucine, and isoleucine biosynthesis; and pantothenate and CoA biosynthesis were also significantly enriched in type I CRA (all FDR adjusted *p* < 0.05), with their corresponding matched differential metabolites being l-valine. Additionally, the pathways of alpha-linolenic acid metabolism and biosynthesis of unsaturated fatty acids were significantly enriched in type I CRA (all FDR adjusted *p* < 0.05), with their corresponding matched differential metabolites being linolenic acid. The pathway of purine metabolism was significantly enriched in type III CRA (FDR adjusted *p* < 0.05), with its corresponding matched differential metabolites being xanthosine and inosine. Furthermore, the pathways of folate biosynthesis, nicotinate and nicotinamide metabolism and pyrimidine metabolism were significantly enriched in type III CRA (all FDR adjusted *p* < 0.05), with their corresponding matched differential metabolites being 7,8-dihydropteroic acid, nicotinic acid and uridine. Moreover, the differential metabolites that matched with the pathways of phenylalanine, tyrosine, and tryptophan biosynthesis; phenylalanine metabolism; alpha-linolenic acid metabolism; nicotinate and nicotinamide metabolism; sphingolipid metabolism; tryptophan metabolism; tyrosine metabolism; glycerophospholipid metabolism; pyrimidine metabolism; and purine metabolism demonstrated a significant contribution to these pathways, with impact values greater than zero.

Table S14 shows that in the type I CRC subgroup, *Bacillus* had significant negative correlations with allolithocholic acid, DG(22:6–2OH/0:0/20:0), dodecanedioic acid, glutaric acid, inosine, lithocholic acid, methylglutaric acid, and PA(20:4-OH/i-22:0); *Family XIII AD3011 group* had significant negative correlations with *N*,*N*,*N*-trimethyl-l-alanyl-l-proline betaine and N1-acetylspermidine; *Lachnospiraceae.incertae sedis* had a significant negative correlation with deoxycholylproline; and *Odoribacter* had a significant negative correlation with PA(20:5–3OH/10:0) (all FDR adjusted *p* < 0.05). *Actinomyces* had a significant positive correlation with l-valine; *Alistipes* had a significant positive correlation with methylglutaric acid; *Bacteroides* had a significant positive correlation with tryptamine; and *Eggerthella* had a significant positive correlation with asparaginyl-valine (all FDR adjusted *p* < 0.05). In the type III CRC subgroup, only *Christensenellaceae R7 group* had a significant negative correlation with ursodeoxycholic acid 3-sulfate (FDR adjusted *p* = 0.039). No significant correlations were observed between differential metabolites and differential bacterial genera in either subgroup of the CRA group.

## 4. Discussion

In this study, we identified two distinct dietary patterns and three gut microbiota enterotypes among Han Chinese individuals with colorectal neoplasms or controls. We found that a healthy dietary pattern, characterized by high consumption of vegetables, fruits, milk, and yogurt, was associated with a reduced risk of CRC, especially in individuals with type I and type II gut microbiota enterotypes, which were dominated by *Bacteroides* and *Lachnoclostridium* or *Bacteroides* and *Faecalibacterium*, respectively. Fruit consumption was the main contributor to this protective effect. We did not find any associations between a healthy dietary pattern and the risk of CRA or between a high-fat dietary pattern and the risk of colorectal neoplasms. These findings partially supported our hypothesis that different dietary patterns could differentially affect the risk of developing colorectal tumors in individuals with various gut microbiota enterotypes.

We first discussed how dietary patterns influence the risk of colorectal neoplasms. We found that a healthy dietary pattern was inversely associated with CRC risk. This is consistent with previous studies that have reported protective effects of a prudent diet [3], a Mediterranean diet [4], or an Asian diet [5] on CRC risk. These diets share some common features with our healthy dietary pattern, such as high intake of plant-based foods and low intake of red meat and processed meat. The beneficial effects of these foods on CRC risk may be attributed to their high content of antioxidants, phytochemicals, fiber, calcium, and probiotics, which can modulate oxidative stress, inflammation, DNA damage, apoptosis, and immune response in the colon [25]. Similar dietary patterns have also been described by other authors in other studies [9,26], indicating that they may have universal effects on colorectal neoplasm development. On the other hand, we did not find any association between a high-fat dietary pattern, marked by high consumption of pickled, fried, and red meat products, and low intake of healthy foods, and CRC risk. This is somewhat surprising given that several studies have linked a Western diet, characterized by high intake of red meat, processed meat, refined grains, and sugar-sweetened beverages, to increased CRC risk [2,25]. The discrepancy may be due to the differences in the definition and measurement of dietary patterns, as well as the potential confounding or modifying effects of other lifestyle factors. Moreover, we did not find any associations between dietary patterns and CRA risk. This may suggest that dietary factors have a stronger impact on the progression than the initiation of colorectal tumors [27,28]. Alternatively, this may reflect the limited statistical power to detect small effects due to the relatively small sample size and low exposure contrast in our study.

We then explored whether gut microbiota mediates or modifies the relationship between dietary patterns and colorectal neoplasm risk. Gut microbiota, a complex ecosystem of microorganisms in the human gastrointestinal tract, affects various aspects of human health and disease [29]. Gut microbiota influences CRC pathogenesis through host metabolism, immunity, inflammation, and genotoxicity [30]. CRC patients have a shifted gut microbiota compared to healthy controls [7], with different enterotypes based on predominant bacterial genera [11]. These enterotypes reflect varied metabolic capacities and responses to diet [8]. We hypothesized that dietary patterns affect colorectal tumor risk differently in individuals with various enterotypes. We tested this hypothesis by categorizing 250 cases into three enterotypes (subtypes) based on OTU abundance profiles using DMM. We found that a higher adherence to a healthy dietary pattern reduced the risk of type I CRC (dominated by *Bacteroides* and *Lachnoclostridium*) and type II CRC (dominated by *Bacteroides* and *Faecalibacterium*), but not type III CRC (characterized by *Prevotella 9*). This suggests that enterotypes modify the diet-CRC risk association. A possible explanation is that enterotypes metabolize dietary components differently into compounds that affect CRC development. For example, *Bacteroides*, *Lachnoclostridium* and *Faecalibacterium* produce SCFAs from fiber fermentation [31,32], which have anti-inflammatory and anti-tumorigenic effects in the colon [33,34]. *Prevotella 9* produces trimethylamine (TMA) from choline and carnitine [35,36], which converts to trimethylamine *N*-oxide (TMAO) in the liver and promotes inflammation and oxidative stress in the colon [37]. Therefore, a healthy dietary pattern benefits CRC risk more in individuals with type I and type II enterotypes than in those with type III enterotype. We also observed that a healthy dietary pattern lowered the risk of colorectal neoplasms in the distal colon and rectum, but not in the proximal colon. This may be due to higher exposure of the distal colon and rectum to diet and microbial metabolites than the proximal colon [38]. Moreover, different molecular pathways and genetic alterations may be involved in the development of colorectal tumors in different locations [39]. In addition to these general mechanisms, we also explored how specific food items within a healthy dietary pattern, such as fruit, may influence CRC risk by modulating gut microbiota.

Among the food items within a healthy dietary pattern, we found that fruit consumption was the main factor that lowered CRC risk in individuals with type I and type II gut microbiota enterotypes. This may be explained by several mechanisms. First, fruit is rich in antioxidants, such as vitamin C and polyphenols, which can scavenge reactive oxygen species and reduce oxidative stress in the gut [40]. Second, fruit has anti-inflammatory properties that can modulate immune responses and cytokine production in the gut [41]. Third, fruit can serve as a prebiotic substrate for beneficial bacteria, such as *Bacteroides* and *Faecalibacterium*, which can produce SCFAs and other metabolites with anticancer effects [42].

To further elucidate the mechanisms underlying the differential associations of a healthy dietary pattern with CRC risk by gut microbiota enterotypes, we compared the diversity, composition, and function of gut microbiota between different tumor subtypes. We found that type I and type III CRC subtypes had distinct microbial community profiles, with significant differences in alpha-diversity and beta-diversity. Alpha-diversity denotes the richness and evenness of microbial species within a sample, whereas beta-diversity reflects the similarity or dissimilarity of microbial communities across samples. Lower alpha-diversity and higher beta-diversity indicate a dysbiosis between beneficial and harmful bacteria that may increase CRC risk by disrupting the balance of host-microbe interactions [43]. We also identified 44 bacterial genera that differed significantly in abundance between type I and type III CRC subtypes. Some of these genera have been previously reported to be associated with CRC risk, such as *Escherichia shigella* [44], *Bifidobacterium* [45], *Bacteroides* [46], *Prevotella 9* [47], *Alistipes* [48], and *Odoribacter* [49]. These bacteria may affect CRC development by producing or modulating various metabolites with pro- or anti-carcinogenic effects, such as SCFAs, TMA/TMAO, bile acids, secondary bile acids, polyamines, nitrosamines, and hydrogen sulfide [50].

To investigate the functional differences of gut microbiota between type I and type III CRC subtypes, we performed metabolomic profiling using UHPLC-QTOFMS. We identified 50 metabolites that differed significantly in abundance between these subtypes. These metabolites were involved in several metabolic pathways that have been implicated in CRC pathogenesis, such as fatty acid degradation [34], tryptophan metabolism [51], primary bile acid biosynthesis [52], purine metabolism [53], thiamine metabolism [54], sphingolipid metabolism [55], and nicotinate and nicotinamide metabolism [56]. We also assessed the correlations between differential metabolites and differential bacterial genera in these subtypes. We found several significant correlations that may reflect the interactions among diet, gut microbiota, and host metabolism. For example, we found a positive correlation between *Bacteroides* and tryptamine, a metabolite derived from tryptophan that can induce apoptosis and inhibit proliferation of CRC cells [57]. We also found a negative correlation between *Bacillus* and allolithocholic acid, a secondary bile acid that can promote inflammation and DNA damage in the colon [58]. These correlations suggest that some bacteria may modulate the production or degradation of certain metabolites that influence CRC risk.

Our study has several strengths. First, we used ESEM to identify dietary patterns based on eight food items that are commonly consumed in China. ESEM is a novel technique that allows for cross-loadings between factors and indicators, providing a more realistic representation of dietary patterns than traditional methods such as PCA or FA [19]. Second, we used DMM to classify colorectal neoplasm cases into gut microbiota enterotypes based on their OTU abundance profiles. DMM is a probabilistic method for community typing of microbial data that can infer the optimal number of community types [20]. Third, we used UHPLC-QTOFMS to perform metabolomic profiling of fecal samples from colorectal neoplasm cases. UHPLC-QTOFMS is a powerful technique that can detect a wide range of metabolites with high sensitivity and accuracy.

However, our study also has some limitations. First, we did not perform sample size calculation prior to the study, nor did we calculate the Beta error for each analysis after the study. This was mainly due to the complexity and novelty of our study design and methods, which involved multiple comparisons, data-driven approaches, and high-throughput techniques. Moreover, there were no prior data available in the literature on the relationship between dietary patterns, gut microbiota, and colorectal neoplasms that we could use as references for sample size calculation. The sample size of our study was comparable to or larger than most of the published studies on gut microbiota and colorectal neoplasms before 2015, which rarely exceeded 100 cases. However, we acknowledge that our sample size may not be sufficient to detect small or moderate effects, especially for some subgroups or subtypes with low prevalence. Therefore, our results should be interpreted with caution and validated in larger and more diverse cohorts. Future studies may also consider using simulation methods or Bayesian approaches to estimate sample size or statistical power for complex and exploratory studies similar to ours. Second, we only assessed eight food items in our food frequency questionnaire, which may not capture the full range of dietary intake and diversity. This may limit the accuracy and generalizability of our results. Therefore, our results should be interpreted with caution and validated by future studies with more comprehensive dietary assessments. Moreover, we did not collect information on the types and quantities of fruit consumed by participants, which may affect their impact on gut microbiota and CRC risk. Future studies should also investigate how different types and amounts of fruit may modulate gut microbiota and CRC risk in relation to individual and environmental factors. Third, our study population was limited to Han Chinese and may not be representative of other populations with different genetic and environmental backgrounds. Previous studies have shown that gut microbiota composition and function can vary across different ethnic groups and geographic regions [59,60]. Therefore, our findings may not be generalizable to other populations and should be confirmed by future studies with more diverse samples. Fourth, our gut microbiota analysis was based on 16S rDNA sequencing, which only provides information on bacterial taxa but not on their functions or interactions. Metabolomics analysis can partially reflect the functional potential of gut microbiota, but it may also be influenced by other factors such as host metabolism and environmental exposure. Fifth, our study was cross-sectional in nature, which precludes any causal inference between dietary patterns, gut microbiota enterotypes, and colorectal neoplasm risk. Longitudinal studies are needed to establish the temporal sequence and direction of these associations.

## 5. Conclusions

In conclusion, our study suggests that a healthy dietary pattern, rich in fruits, vegetables, milk, and yogurt, is associated with a decreased risk of CRC, particularly in individuals with type I and type II gut microbiota enterotypes. These enterotypes are characterized by distinct microbial compositions and functions that may modulate the effects of diet on CRC development. Fruit consumption was the main contributor to this protective effect. Our findings provide novel insights into the relationship between dietary patterns, gut microbiota, and colorectal neoplasm risk, and may have implications for future prevention strategies. However, further studies with larger sample sizes, more comprehensive dietary assessments, more advanced gut microbiota and metabolomic analyses, and longitudinal designs are needed to confirm and extend our results.

## Figures and Tables

**Table 1:** Characteristics of the study population.

Variable	Colorectal Cancer (*n* = 130)	Colorectal Adenoma (*n* = 120)	Control Group (*n* = 160)
Healthy dietary pattern ^a^			
Median	−0.21	0.02	0.22
Range	−2.37 to 1.93	−2.09 to 1.93	−1.62 to 1.93
High-fat dietary pattern ^a^			
Median	−0.2	−0.01	−0.04
Range	−1.77 to 3.02	−1.77 to 2.89	−1.42 to 2.89
Age, years			
Mean (SD)	60.54 (9.84)	59.06 (10.11)	57.98 (8.82)
Range	40 to 88	40 to 84	40 to 79
Sex, No. (%)			
Female	65 (50.0)	49 (40.8)	80 (50.0)
Male	65 (50.0)	71 (59.2)	80 (50.0)
Education degree, No. (%)			
Illiteracy	15 (12.0)	5 (4.2)	6 (4.0)
Primary	20 (15.0)	22 (18.3)	23 (14.0)
Middle	79 (61.0)	65 (54.2)	101 (63.0)
High	16 (12.0)	28 (23.3)	30 (19.0)
Physical activity, No. (%)			
Sedentary	22 (16.9)	30 (25.0)	29 (18.0)
Mild	53 (40.8)	55 (46.0)	75 (47.0)
Moderate	31 (23.8)	25 (21.0)	41 (26.0)
Severe	24 (18.5)	10 (8.0)	15 (9.0)
Smoking, No. (%)			
No	91 (70.0)	75 (62.5)	126 (79.0)
Yes	39 (30.0)	45 (37.5)	34 (21.0)
Mean (SD), pack-years	8.38 (15.05)	9.08 (15.34)	5.86 (14.37)
Range, pack-years	0 to 60	0 to 100	0 to 70
Drinking, No. (%)			
No	98 (75.0)	87 (72.5)	133 (83.0)
Yes	32 (25.0)	33 (27.5)	27 (17.0)
Body mass index ^b^, kg/m^2^			
Mean (SD)	23.59 (3.09)	24.03 (3.33)	23.87 (3.28)
Range	17.02 to 33.33	15.43 to 32.24	16.53 to 35.16

Abbreviations: SD, standard deviation. ^a^ A healthy dietary pattern is characterized by a high intake of vegetables, fruits, milk, and yogurt, and a low intake of high-fat food products. Conversely, a high-fat dietary pattern is characterized by a high intake of pickled, fried, and red meat products, and a low intake of healthy foods. The median and range of factor scores are displayed in the table. ^b^ Body mass index: weight (kg)/height (m)^2^.

**Table 2:** Dietary patterns and risk of colorectal neoplasms, overall and subclassified by gut microbiota enterotypes ^a^.

Group	Healthy Pattern	*p* ^b^	*p*_heterogeneity_ ^c^	High-Fat Pattern	*p* ^b^	*p*_heterogeneity_ ^c^
Control (*N* = 160)						
Median (Range)	0.22 (−1.62 to 1.93)			−0.04 (−1.42 to 2.89)		
All colorectal cancer (*N* = 130)						
Median (Range)	−0.21 (−2.37 to 1.93)	<0.001		−0.20 (−1.77 to 3.02)	0.997	
Multivariable-adjusted OR (95% CI) ^d^	0.62 (0.48 to 0.81)	0.001		1.06 (0.83 to 1.35)	0.631	
Type I colorectal cancer (*N* = 68)			0.345			0.601
Median (Range)	−0.18 (−1.83 to 1.93)	0.007	(type I vs. II)	−0.20 (−1.77 to 3.02)	0.868	(type I vs. II)
Multivariable-adjusted OR (95% CI) ^d^	0.66 (0.48 to 0.89)	0.006		1.04 (0.77 to 1.40)	0.801	
Type II colorectal cancer (*N* = 48)			0.026			0.132
Median (Range)	−0.82 (−2.37 to 1.72)	<0.001	(type II vs. III)	−0.23 (−1.53 to 2.64)	0.581	(type II vs. III)
Multivariable-adjusted OR (95% CI) ^d^	0.42 (0.29 to 0.62)	<0.001		0.94 (0.67 to 1.32)	0.940	
Type III colorectal cancer (*N* = 14)			0.037			0.225
Median (Range)	0.02 (−1.40 to 1.93)	0.592	(type I vs. III)	−0.08 (−0.81 to 2.65)	0.128	(type I vs. III)
Multivariable-adjusted OR (95% CI) ^d^	0.94 (0.52 to 1.69)	0.828		1.44 (0.88 to 2.37)	0.149	
All colorectal adenoma (*N* = 120)						
Median (Range)	0.02 (−2.09 to 1.93)	0.099		−0.01 (−1.77 to 2.89)	0.668	
Multivariable-adjusted OR (95% CI) ^d^	0.83 (0.65 to 1.07)	0.146		1.01 (0.79 to 1.29)	0.957	
Type I colorectal adenoma (*N* = 50)			0.860			0.830
Median (Range)	−0.11 (−1.85 to 1.93)	0.042	(type I vs. II)	−0.11 (−1.77 to 2.89)	0.839	(type I vs. II)
Multivariable-adjusted OR (95% CI) ^d^	0.72 (0.51 to 1.01)	0.059		0.99 (0.72 to 1.35)	0.930	
Type II colorectal adenoma (*N* = 52)			0.010			0.623
Median (Range)	0.00 (−2.09 to 1.72)	0.082	(type II vs. III)	−0.01 (−1.77 to 2.73)	0.869	(type II vs. III)
Multivariable-adjusted OR (95% CI) ^d^	0.70 (0.49 to 1.00)	0.050		1.00 (0.71 to 1.41)	0.988	
Type III colorectal adenoma (*N* = 18)			0.007			0.740
Median (Range)	0.93 (−0.86 to 1.93)	0.118	(type I vs. III)	−0.01 (−0.56 to 1.62)	0.147	(type I vs. III)
Multivariable-adjusted OR (95% CI) ^d^	1.47 (0.84 to 2.56)	0.178		1.23 (0.74 to 2.04)	0.436	

Abbreviations: CI, confidence interval; OR, odds ratio. ^a^ The colorectal cancer and colorectal adenoma groups were divided into three enterotypes (or subtypes), labeled as type I, type II, and type III, based on their gut microbiota profiles, using the Dirichlet multinomial mixture model. ^b^ The *p* values represent the comparison between the case group (colorectal cancer or adenoma, including their subtypes) and the control group, either in the univariate or multivariate analysis. ^c^ The *p*_heterogeneity_ value represents a test for heterogeneity to assess whether there is a significant difference in the association between dietary patterns and the risk of different subtypes of colorectal tumors. ^d^ The multivariable odds ratio (OR) was adjusted for potential risk factors with *p*-values less than 0.1 in the univariate analysis.

**Table 3:** Dietary patterns and risk of colorectal neoplasms, overall and subclassified by gut microbiota enterotypes ^a^.

Group	Healthy Pattern			*p* ^b^	*p*_heterogeneity_ ^c^	High-Fat Pattern			*p* ^b^	*p*_heterogeneity_ ^c^
	Quartile 1	Quartile 2	Quartile 3			Quartile 1	Quartile 2	Quartile 3		
Control (*N* = 160)										
No. (%)	56 (35.0)	52 (32.5)	52 (32.5)			59 (36.9)	51 (31.9)	50 (31.2)		
All colorectal cancer (*N* = 130)										
No. (%)	80 (61.5)	24 (18.5)	26 (20.0)	<0.001		53 (40.8)	36 (27.7)	41 (31.5)	0.704	
Multivariable-adjusted OR (95% CI) ^d^	1 (Referent)	0.36 (0.19 to 0.66)	0.38 (0.2 to 0.71)	0.001		1 (Referent)	0.80 (0.45 to 1.44)	0.99 (0.55 to 1.76)	0.925	
Type I colorectal cancer (*N* = 68)					0.644					0.636
No. (%)	39 (57.4)	12 (17.6)	17 (25.0)	0.006	(type I vs. II)	31 (45.6)	15 (22.1)	22 (32.3)	0.283	(type I vs. II)
Multivariable-adjusted OR (95% CI) ^d^	1 (Referent)	0.40 (0.18 to 0.86)	0.52 (0.25 to 1.11)	0.054		1 (Referent)	0.62 (0.30 to 1.31)	0.97 (0.48 to 1.95)	0.824	
Type II colorectal cancer (*N* = 48)					0.046					0.139
No. (%)	35 (72.9)	7 (14.6)	6 (12.5)	<0.001	(type II vs. III)	20 (41.7)	15 (31.2)	13 (27.1)	0.803	(type II vs. III)
Multivariable-adjusted OR (95% CI) ^d^	1 (Referent)	0.21 (0.09 to 0.52)	0.19 (0.07 to 0.48)	<0.001		1 (Referent)	0.84 (0.39 to 1.83)	0.69 (0.31 to 1.55)	0.369	
Type III colorectal cancer (*N* = 14)					0.049					0.219
No. (%)	6 (42.9)	5 (35.7)	3 (21.4)	0.683	(type I vs. III)	2 (14.3)	6 (42.9)	6 (42.9)	0.236	(type I vs. III)
Multivariable-adjusted OR (95% CI) ^d^	1 (Referent)	0.8 (0.22 to 2.85)	0.51 (0.12 to 2.19)	0.722		1 (Referent)	3.22 (0.61 to 16.91)	3.26 (0.62 to 17.18)	0.179	
All colorectal adenoma (*N* = 120)										
No. (%)	59 (49.2)	26 (21.7)	35 (29.2)	0.039		45 (37.5)	36 (30.0)	39 (32.5)	0.942	
Multivariable-adjusted OR (95% CI) ^d^	1 (Referent)	0.48 (0.27 to 0.88)	0.67 (0.38 to 1.18)	0.120		1 (Referent)	0.90 (0.50 to 1.60)	0.97 (0.54 to 1.73)	0.901	
Type I colorectal adenoma (*N* = 50)					0.643					0.927
No. (%)	27 (54.0)	12 (24.0)	11 (22.0)	0.056	(type I vs. II)	21 (42.0)	12 (24.0)	17 (34.0)	0.565	(type I vs. II)
Multivariable-adjusted OR (95% CI) ^d^	1 (Referent)	0.48 (0.22 to 1.06)	0.44 (0.20 to 0.99)	0.033		1 (Referent)	0.62 (0.27 to 1.39)	0.88 (0.42 to 1.88)	0.710	
Type II colorectal adenoma (*N* = 52)					0.030					0.438
No. (%)	26 (50.0)	12 (23.1)	14 (26.9)	0.147	(type II vs. III)	22 (42.3)	13 (25.0)	17 (32.7)	0.624	(type II vs. III)
Multivariable-adjusted OR (95% CI) ^d^	1 (Referent)	0.46 (0.20 to 1.05)	0.50 (0.23 to 1.09)	0.068		1 (Referent)	0.58 (0.26 to 1.31)	0.94 (0.44 to 1.99)	0.804	
Type III colorectal adenoma (*N* = 18)					0.013					0.477
No. (%)	6 (33.3)	2 (11.1)	10 (55.6)	0.085	(type I vs. III)	2 (11.1)	11 (61.1)	5 (27.8)	0.028	(type I vs. III)
Multivariable-adjusted OR (95% CI) ^d^	1 (Referent)	0.30 (0.06 to 1.62)	1.63 (0.53 to 4.98)	0.295		1 (Referent)	6.38 (1.33 to 30.7)	2.67 (0.49 to 14.55)	0.346	

Abbreviations: CI, confidence interval; OR, odds ratio. ^a^ The colorectal cancer and colorectal adenoma groups were divided into three enterotypes (or subtypes), labeled as type I, type II, and type III, based on their gut microbiota profiles, using the Dirichlet multinomial mixture model. ^b^ The *p* values represent the comparison between the case group (colorectal cancer or adenoma, including their subtypes) and the control group, either in the univariate or multivariate analysis. In the multivariate analysis, *p* values were determined by the linear trend test, which utilized multivariable logistic regression. ^c^ The *p*_heterogeneity_ value represents a test for heterogeneity to assess whether there is a significant difference in the association between dietary patterns and the risk of different subtypes of colorectal tumors. ^d^ The multivariable odds ratio (OR) was adjusted for potential risk factors with *p*-values less than 0.1 in the univariate analysis.

## Data Availability

Data supporting the findings of this study are available within the paper and its supplementary information files. Further information is available from the corresponding authors upon request.

## Supplementary Materials

The following supporting information can be downloaded at: https://www.mdpi.com/article/10.3390/nu15132940/s1. Table S1: Inclusion and exclusion criteria for the study population; Table S2: Statistical parameters and methods for each analysis; Table S3: Geomin-rotated factor loading matrix for dietary patterns; Table S4: Characteristics of all participants by healthy and high-fat dietary score tertiles; Table S5: Top four food items (ranked by factor loadings) in the healthy dietary pattern and risk of colorectal neoplasms, overall and subclassified by gut microbiota enterotypes; Table S6: Healthy dietary pattern score and risk of colorectal neoplasms stratified by lesion site, overall and subclassified by gut microbiota enterotypes; Table S7: Post-hoc pairwise comparisons of alpha-diversity indices in CRC and CRA subgroups using Dunn’s test for multiple comparisons; Table S8: Genera with significant differential abundance in both CRC and CRA group comparisons between type I and type III subtypes; Table S9: Differential abundance of metabolites between type I and type III subtypes in CRC group; Table S10: Differential abundance of metabolites between type I and type III subtypes in CRA group; Table S11: Metabolites with significant differential abundance in both CRC and CRA group comparisons between type I and type III subtypes; Table S12: Pathway analysis results for differential metabolites between type I and type III subtypes in colorectal cancer group; Table S13: Pathway analysis results for differential metabolites between type I and type III subtypes in colorectal adenoma group; Table S14: Correlation between differential metabolites and differential bacterial genera in type I CRC subgroup; Figure S1: Combined visualization of taxon representation, scatterplot matrix, and Laplace approximate log-likelihood values for the Dirichlet Multinomial mixture model; Figure S2: Heatmap of the top 30 taxa with the largest differences between the baseline model and the best Dirichlet multinomial mixture model; Figure S3: Principal coordinate analysis (PCoA) plots based on Bray-Curtis distances depict the three distinct microbial community groups (type I, type II, and type III) in colorectal neoplasm samples; Figure S4: Principal coordinate analysis (PCoA) plots based on Manhattan distances illustrate the distinct metabolic profiles between subtypes (type I and type III) in colorectal neoplasm samples; Figure S5: Overview of pathway analysis for differential metabolites between type I and type III subtypes in (A) colorectal cancer and (B) colorectal adenoma groups.
