# Supplementary material for: Gut Microbiota Enterotypes Mediate the Effects of Dietary Patterns on Colorectal Neoplasm Risk in a Chinese Population
Source: Nutrients. 2023 Jun 28;15(13):2940. doi: 10.3390/nu15132940 (PMC10346730; doi:10.3390/nu15132940)
Supplement: Supplementary file 1 [file nutrients-15-02940-s001.zip › nutrients-2442108-supplementary.pdf]

Supplementary Materials

Supplementary Table

Table S1. Inclusion and exclusion criteria for the study population.

Inclusion criteria	Exclusion criteria
Han Chinese individuals aged 40 or older who were recommended to undergo colonoscopy at Changhai Hospital (Shanghai, China) between 2015 and 2016	Incomplete colonoscopies
	Antibiotic use in the past 6 months
	Pregnancy
	A history of colorectal neoplasm, inflammatory bowel disease, hereditary polyposis syndromes, or any other type of cancer
	A family history of colorectal cancer in a first- or second-degree relative
	A family history of colorectal adenoma or familial hereditary syndrome, including familial adenomatous polyposis, hereditary nonpolyposis colorectal cancer, Turcot syndrome, Oldfield syndrome, and juvenile polyposis syndrome in a first-degree relative under 60 years of age
	Prior chemotherapy, radiation therapy, or colorectal surgery

Table S2. Statistical parameters and methods for each analysis.

Analysis	Statistical parameters	Statistical methods
Identification of dietary patterns	Factor loadings, eigenvalues	Exploratory structural equation modeling (ESEM) with oblique rotation
Relationship between dietary patterns and risk of colorectal neoplasms	Odds ratios (ORs), confidence intervals (CIs), P values	Binary logistic backward stepwise regression analysis
Relationship between dietary patterns and risk of colorectal neoplasm subtypes	ORs, CIs, P values, $P_{heterogeneity}$ values	Dirichlet multinomial mixture model (DMM) for community typing, Wald test for heterogeneity, binary logistic backward stepwise regression analysis
Gut microbiota composition analysis between subgroups	Alpha-diversity indices, beta-diversity distances, P values, logarithmic linear discriminant analysis (LDA) score	Kruskal-Wallis test, Dunn's test, principal coordinates analysis (PCoA), permutational multivariate analysis of variance (PERMANOVA), LDA effect size (LEfSe) method
Metabolomics-based analysis of gut microbiota functional differences between subgroups	Metabolite features, logarithmic LDA scores, P values, pathway enrichment scores, pathway impact values, correlation coefficients	Logarithmic transformation, Z-score standardization, PCoA, LEfSe method, Kyoto Encyclopedia of Genes and Genomes (KEGG) mapping, pathway enrichment analysis, pathway topology analysis, Spearman's correlation coefficient

Table S3. Geomin-rotated factor loading matrix for dietary patterns.

Food item [a]	Healthy pattern	High-fat pattern
Vegetable	0.365	
Fruit	0.746	
Milk	0.592	
Yoghourt	0.611	
Pickle		0.660
Fry		0.831
Red meat		0.426

[a] Only food items with structure coefficients greater than 0.30 are displayed. Structure coefficients, also known as factor loadings, refer to the Geomin-rotated factor loading matrix.

Table S4. Characteristics of all participants by healthy and high-fat dietary score tertiles.

Characteristic	Healthy pattern		High-fat pattern	
	Q1	Q3	Q1	Q3
**Age, years (mean)**	58.9	60.3	61.0	57.1
**Sex, man (%)**	56.4	48.7	44.6	61.5
Education degree (%)				
Illiteracy	8.7	1.8	8.3	4.6
Primary	21.0	9.7	21.0	14.6
Middle	59.0	61.1	59.9	60.8
High	11.3	27.4	10.8	20.0
Physical activity (%)				
Sedentary	18.5	26.5	17.2	19.2
Mild	35.4	50.4	42.7	43.1
Moderate	27.7	20.4	24.8	24.6
Severe	18.5	2.7	15.3	13.1
**Smoking (%)**	36.9	20.4	24.8	33.1
**Smoking, pack-years (mean)**	10.7	4.6	6.0	9.7
**Drinking (%)**	26.2	15.9	16.6	30.8
Body mass index [a], kg/m^2 (mean)	23.7	23.7	23.0	24.7

Abbreviations: SD, standard deviation.

[a] Body mass index: weight (kg)/height $(m)^2$.

Table S5. Top four food items (ranked by factor loadings) in the healthy dietary pattern and risk of colorectal neoplasms, overall and subclassified by gut microbiota enterotypes [a]

Group	Consumption frequency		P [b]	$P_{heterogeneity}$ [c]
	Occasional	regular		
**Vegetable**				
Control (N=160), No. (%)	4 (2.5)	156 (97.5)		
All colorectal neoplasm (N=250), No. (%)	20 (8.0)	230 (92.0)	0.021	
Multivariable-adjusted OR (95% CI) [d]	1 (referent)	0.39 (0.13 to 1.18)	0.094	
Type I colorectal neoplasm (N=118), No. (%)	8 (6.8)	110 (93.2)	0.083	0.372
Multivariable-adjusted OR (95% CI) [d]	1 (referent)	0.41 (0.12 to 1.40)	0.153	(type I vs II)
Type II colorectal neoplasm (N=100), No. (%)	10 (10.0)	90 (90.0)	0.009	0.564
Multivariable-adjusted OR (95% CI) [d]	1 (referent)	0.26 (0.08 to 0.86)	0.028	(type II vs III)
Type III colorectal neoplasm (N=32), No. (%)	2 (6.3)	30 (93.8)	0.311	0.981
Multivariable-adjusted OR (95% CI) [d]	1 (referent)	0.36 (0.06 to 2.12)	0.258	(type I vs III)
**Fruit**				
Control (N=160), No. (%)	42 (26.3)	118 (73.8)		
All colorectal neoplasm (N=250), No. (%)	103 (41.2)	147 (58.8)	0.002	
Multivariable-adjusted OR (95% CI) [d]	1 (referent)	0.55 (0.36 to 0.86)	0.009	
Type I colorectal neoplasm (N=118), No. (%)	49 (41.5)	69 (58.5)	0.007	0.314
Multivariable-adjusted OR (95% CI) [d]	1 (referent)	0.51 (0.31 to 0.85)	0.010	(type I vs II)
Type II colorectal neoplasm (N=100), No. (%)	48 (48.0)	52 (52.0)	<0.001	0.006
Multivariable-adjusted OR (95% CI) [d]	1 (referent)	0.41 (0.24 to 0.69)	0.001	(type II vs III)
Type III colorectal neoplasm (N=32), No. (%)	6 (18.8)	26 (81.3)	0.371	0.027
Multivariable-adjusted OR (95% CI) [d]	1 (referent)	1.54 (0.58 to 4.03)	0.385	(type I vs III)
**Milk**				
Control (N=160), No. (%)	106 (66.3)	54 (33.8)		
All colorectal neoplasm (N=250), No. (%)	175 (70.0)	75 (30.0)	0.425	
Multivariable-adjusted OR (95% CI) [d]	1 (referent)	0.89 (0.57 to 1.40)	0.623	
Type I colorectal neoplasm (N=118), No. (%)	80 (67.8)	38 (32.2)	0.786	0.121
Multivariable-adjusted OR (95% CI) [d]	1 (referent)	0.81 (0.48 to 1.37)	0.430	(type I vs II)
Type II colorectal neoplasm (N=100), No. (%)	77 (77.0)	23 (23.0)	0.065	0.012
Multivariable-adjusted OR (95% CI) [d]	1 (referent)	0.62 (0.35 to 1.09)	0.098	(type II vs III)
Type III colorectal neoplasm (N=32), No. (%)	18 (56.3)	14 (43.8)	0.280	0.142
Multivariable-adjusted OR (95% CI) [d]	1 (referent)	1.56 (0.72 to 3.43)	0.263	(type I vs III)
**Yoghourt**				
Control (N=160), No. (%)	119 (74.4)	41 (25.6)		
All colorectal neoplasm (N=250), No. (%)	203 (81.2)	47 (18.8)	0.101	
Multivariable-adjusted OR (95% CI) [d]	1 (referent)	0.71 (0.43 to 1.16)	0.172	
Type I colorectal neoplasm (N=118), No. (%)	97 (82.2)	21 (17.8)	0.121	0.761
Multivariable-adjusted OR (95% CI) [d]	1 (referent)	0.59 (0.32 to 1.07)	0.084	(type I vs II)
Type II colorectal neoplasm (N=100), No. (%)	82 (82.0)	18 (18.0)	0.153	0.301
Multivariable-adjusted OR (95% CI) [d]	1 (referent)	0.66 (0.35 to 1.23)	0.186	(type II vs III)
Type III colorectal neoplasm (N=32), No. (%)	24 (75.0)	8 (25.0)	0.941	0.202
Multivariable-adjusted OR (95% CI) [d]	1 (referent)	1.02 (0.42 to 2.48)	0.966	(type I vs III)

Abbreviations: CI, confidence interval; OR, odds ratio.

[a] Colorectal neoplasms were classified into three gut microbiota enterotypes (or subtypes), designated as type I, type II, and type III, using the Dirichlet multinomial mixture model based on their gut microbiota profiles.

[b] The P values represent the comparison between the case group (colorectal neoplasms, including their subtypes) and the control group, either in the univariate or multivariate analysis.

[c] The $P_{heterogeneity}$ value represents a test for heterogeneity to assess whether there is a significant difference in the association between food items and the risk of different subtypes of colorectal tumors.

[d] The multivariable odds ratio (OR) was adjusted for potential risk factors with P-values less than 0.1 in the univariate analysis.

Table S6. Healthy dietary pattern score and risk of colorectal neoplasms stratified by lesion site, overall and subclassified by gut microbiota enterotypes [a]

Group	Healthy dietary pattern			P^{b}	$P_{heterogeneity}{}^{c}$
	Quartile 1	Quartile 2	Quartile 3		
Control (N=160), No. (%)	56 (35.0)	52 (32.5)	52 (32.5)		
**Proximal colon** [d]					
All colorectal neoplasm (N=86), No. (%)	48 (55.8)	14 (16.3)	24 (27.9)	0.003	
Multivariable-adjusted OR (95% CI) [e]	1 (referent)	0.34 (0.17 to 0.70)	0.51 (0.27 to 0.96)	0.021	
Type I colorectal neoplasm (N=42), No. (%)	20 (47.6)	10 (23.8)	12 (28.6)	0.303	0.398
Multivariable-adjusted OR (95% CI) [e]	1 (referent)	0.59 (0.25 to 1.4)	0.6 (0.26 to 1.37)	0.200	(type I vs II)
Type II colorectal neoplasm (N=37), No. (%)	23 (62.2)	4 (10.8)	10 (27.0)	0.005	0.768
Multivariable-adjusted OR (95% CI) [e]	1 (referent)	0.21 (0.07 to 0.66)	0.47 (0.21 to 1.1)	0.045	(type II vs III)
Type III colorectal neoplasm (N=7), No. (%)	5 (71.4)	0 (0.00)	2 (28.6)	0.041	0.457
Multivariable-adjusted OR (95% CI) [e]	1 (referent)	-	0.43 (0.08 to 2.32)	0.222	(type I vs III)
**Distal colon and rectum** [d]					
All colorectal neoplasm (N=164), No. (%)	91 (55.5)	36 (22.0)	37 (22.6)	0.001	
Multivariable-adjusted OR (95% CI) [e]	1 (referent)	0.43 (0.25 to 0.74)	0.47 (0.27 to 0.80)	0.003	
Type I colorectal neoplasm (N=76), No. (%)	46 (60.5)	14 (18.4)	16 (21.1)	0.001	0.826
Multivariable-adjusted OR (95% CI) [e]	1 (referent)	0.38 (0.18 to 0.8)	0.44 (0.21 to 0.91)	0.014	(type I vs II)
Type II colorectal neoplasm (N=63), No. (%)	38 (60.3)	15 (23.8)	10 (15.9)	0.002	0.002
Multivariable-adjusted OR (95% CI) [e]	1 (referent)	0.43 (0.21 to 0.88)	0.3 (0.14 to 0.67)	0.002	(type II vs III)
Type III colorectal neoplasm (N=25), No. (%)	7 (28.0)	7 (28.0)	11 (44.0)	0.525	0.002
Multivariable-adjusted OR (95% CI) [e]	1 (referent)	0.96 (0.31 to 2.98)	1.65 (0.59 to 4.62)	0.319	(type I vs III)

Abbreviations: CI, confidence interval; OR, odds ratio.

[a] Colorectal neoplasms were classified into three gut microbiota enterotypes (or subtypes), designated as type I, type II, and type III, using the Dirichlet multinomial mixture model based on their gut microbiota profiles.

[b] The P values represent the comparison between the case group (colorectal neoplasms, including their subtypes) and the control group, either in the univariate or multivariate analysis. In the multivariate analysis, P values were determined by the linear trend test, which utilized multivariable logistic regression.

[c] The $P_{heterogeneity}$ value represents a test for heterogeneity to assess whether there is a significant difference in the association between the healthy pattern score and the risk of different subtypes of colorectal tumors.

[d] The proximal colon was defined as the caecum, ascending colon, hepatic flexure, transverse colon, and splenic flexure, while the distal colon was defined as

the descending colon, sigmoid colon, and rectosigmoid junction.

[e] The multivariable odds ratio (OR) was adjusted for potential risk factors with P-values less than 0.1 in the univariate analysis.

Table S7. Post-hoc pairwise comparisons of alpha-diversity indices in CRC and CRA subgroups using Dunn's test for multiple comparisons [a]

Subgroup statistics	Colorectal cancer				Colorectal adenoma			
	Chao	ACE	Shannon	Simpson	Chao	ACE	Shannon	Simpson
Type I								
Median	259.50	268.50	2.94	0.11	235.50	239.50	2.86	0.11
Q1, Q3	217.00, 299.75	218.75, 307.00	2.54, 3.37	0.07, 0.17	187.75, 264.50	200.00, 278.75	2.55, 3.10	0.09, 0.16
Type II								
Median	385.00	379.00	3.63	0.07	379.50	371.00	3.65	0.06
Q1, Q3	332.50, 437.00	337.50, 432.50	3.35, 3.82	0.05, 0.09	335.00, 419.00	335.00, 438.50	3.35, 3.90	0.05, 0.09
Type III								
Median	356.00	363.00	3.33	0.10	360.00	348.50	3.09	0.10
Q1, Q3	324.75, 415.00	319.50, 420.50	2.93, 3.45	0.09, 0.12	286.75, 404.50	293.25, 390.25	2.75, 3.57	0.06, 0.15
Type I vs. type II, P value	< 0.001	< 0.001	< 0.001	< 0.001	< 0.001	< 0.001	< 0.001	< 0.001
Type I vs. type III, P value	< 0.001	< 0.001	0.153	0.945	< 0.001	< 0.001	0.046	0.207
Type II vs. type III, P value	0.349	0.375	0.015	0.005	0.222	0.166	0.001	0.002

Abbreviations: ACE, abundance-based coverage estimator; CRA, colorectal adenoma; CRC, colorectal cancer.

[a] Colorectal neoplasms were categorized into three distinct gut microbiota enterotypes (subtypes) – type I, type II, and type III – employing the Dirichlet multinomial mixture model according to their gut microbiota compositions. The median values are presented along with the first quartile (Q1) and third quartile (Q3) for each subtype.

Table S8. Genera with significant differential abundance in both CRC and CRA group comparisons between type I and type III subtypes [a]

Genus_Subtype	Colorectal cancer			Colorectal adenoma		
	Logarithm value	LDA value	P value	Logarithm value	LDA value	P value
Type I						
Bacteroides	5.48	5.00	0.001	5.55	5.10	< 0.001
Escherichia Shigella	4.78	4.49	< 0.001	4.96	4.60	< 0.001
Lachnoclostridium	4.75	4.30	0.001	4.74	4.29	< 0.001
Bifidobacterium	4.02	3.71	0.050	3.65	3.34	0.037
Flavonifractor	3.72	3.41	< 0.001	3.50	3.11	< 0.001
Tyzzerella 4	3.65	3.32	0.003	3.68	3.32	0.012
Lachnospiraceae UCG-010	3.65	2.83	0.006	3.55	3.18	< 0.001
Erysipelatoclostridium	2.92	2.63	< 0.001	3.35	3.05	< 0.001
Erysipelotrichaceae.Incertae Sedis	2.71	2.38	< 0.001	2.72	2.51	< 0.001
Pseudomonas	2.66	2.32	0.021	2.09	2.87	0.006
Lactococcus	2.58	2.27	0.001	2.26	2.62	0.047
Lachnospiraceae ND3007 group	2.70	2.15	0.004	2.82	2.21	0.027
Eggerthella	2.46	2.04	0.001	2.04	2.60	0.001
Type III						
Prevotella 9	5.63	5.28	< 0.001	5.53	5.17	< 0.001
Ruminococcaceae UCG-002	4.11	3.76	< 0.001	4.04	3.75	< 0.001
Coprococcus 2	3.98	3.71	< 0.001	3.09	2.87	0.001
Alistipes	4.33	3.55	0.021	4.34	3.83	0.001
Lachnospiraceae NK4A136 group	3.98	3.48	< 0.001	3.89	3.26	0.001
Eubacterium coprostanoligenes group	3.86	3.45	< 0.001	3.96	3.61	< 0.001
Subdoligranulum	4.10	3.45	0.001	4.06	3.56	< 0.001
Eubacterium ruminantium group	3.60	3.28	< 0.001	3.64	3.38	< 0.001
Alloprevotella	3.56	3.28	0.001	4.25	3.80	0.001

Sutterella	3.99	3.24	0.003	3.85	3.35	0.036
Prevotella 2	3.85	3.22	0.036	4.34	4.09	0.008
Lachnospiraceae.Incertae Sedis	3.56	3.19	< 0.001	3.72	2.93	0.011
Ruminococcus 1	3.58	3.18	< 0.001	3.25	2.74	0.005
Odoribacter	3.72	3.17	< 0.001	3.43	3.07	< 0.001
Ruminococcaceae UCG-005	3.49	3.17	< 0.001	3.55	3.30	< 0.001
Barnesiella	3.59	3.11	0.003	3.17	2.63	0.001
Ruminococcaceae UCG-014	3.45	3.09	< 0.001	3.72	3.50	0.007
Lachnospira	3.68	3.09	< 0.001	3.76	3.08	0.038
Christensenellaceae R 7 group	3.44	3.09	< 0.001	3.40	3.13	< 0.001
Butyricimonas	3.46	2.95	0.003	3.54	3.06	< 0.001
Ruminococcaceae NK4A214 group	3.15	2.75	< 0.001	3.31	3.05	< 0.001
Ruminococcaceae UCG-003	3.29	2.74	< 0.001	3.37	3.04	< 0.001
Holdemanella	2.98	2.64	0.001	2.60	2.45	0.006
Paraprevotella	3.21	2.62	0.002	3.19	2.60	0.003
Ruminococcaceae UCG-010	2.93	2.61	< 0.001	2.61	2.38	0.003
Family XIII AD3011 group	2.88	2.45	0.001	2.71	2.49	< 0.001
Ruminiclostridium 6	2.77	2.42	< 0.001	2.26	2.40	0.001
Coprococcus 3	3.03	2.34	< 0.001	3.26	2.92	< 0.001
Family XIII UCG-001	2.63	2.25	0.001	2.44	2.54	< 0.001
Mitsuokella	2.49	2.20	0.028	3.35	3.00	0.018
Lachnospiraceae FCS020 group	2.53	2.05	< 0.001	2.53	2.35	< 0.001

Abbreviations: CRA, colorectal adenoma; CRC, colorectal cancer; LDA, linear discriminant analysis.

[a] Colorectal neoplasms were classified into three distinct gut microbiota enterotypes (subtypes) - type I, type II, and type III - using the Dirichlet multinomial mixture model based on their gut microbiota compositions. The Logarithm value column displays the logarithm of the highest mean value among the two subtypes for each genus, representing the magnitude of the genus's abundance in the subtype with the highest mean. The LDA value column presents the logarithmic LDA score for each discriminative genus, quantifying the effect size or the degree of separation between subtypes for each genus, with higher LDA scores indicating more significant differences between subtypes. The P value column shows the p-value from the statistical test (e.g., Kruskal-Wallis

test) used to assess the significance of the differences in genus abundance between subtypes, where smaller p-values indicate a higher level of statistical significance.

Table S9. Differential abundance of metabolites between type I and type III subtypes in CRC group [a]

Metabolite_Subtype	Logarithm value	LDA value	P value
Type I			
L-Valine	4.29	3.72	0.031
Chenodeoxycholic acid sulfate	3.95	3.60	0.006
Cholic acid	3.78	3.35	0.006
Allocholic acid	3.65	3.30	0.013
Ursodeoxycholic acid 3-sulfate	3.61	3.22	0.004
Chenodeoxycholic acid 3-sulfate	3.63	3.19	0.029
N,N,N-Trimethyl-L-alanyl-L-proline betaine	3.54	3.09	0.025
3-Oxocholic acid	3.31	2.95	0.004
Oleoylcarnitine	3.22	2.87	<0.001
LysoPA(22:5/0:0)	3.27	2.86	0.003
L-Palmitoylcarnitine	3.20	2.86	<0.001
LysoPC(16:0/0:0)	3.24	2.84	0.022
Leucylproline	3.19	2.78	0.004
Linoleyl carnitine	3.15	2.77	0.038
Oleoylethanolamide	3.24	2.76	0.039
7-Ketodeoxycholic acid	3.02	2.72	0.025
2-Pentenoic acid	3.14	2.62	0.036
LysoPA(18:3/0:0)	2.77	2.47	0.008
Tryptamine	2.79	2.46	0.003
LysoPC(O-16:0/0:0)	2.84	2.44	0.008
Docosa-pentaenoyl carnitine	1.70	2.43	0.048

7-Sulfocholic acid	2.85	2.42	0.028
Phenylalanylglycine	2.12	2.35	0.015
Muricholic acid	2.81	2.35	0.011
Histidylleucine	2.39	2.33	0.003
Cholylasparagine	1.92	2.33	0.014
Tetradecanoylcarnitine	2.58	2.33	<0.001
Deoxycholylproline	2.67	2.32	<0.001
3-Hydroxyoctadecenoylcarnitine	2.58	2.30	<0.001
LysoPA(22:6/0:0)	2.67	2.27	0.002
LysoPE(18:4/0:0)	2.50	2.27	0.001
Pentadecanoylcarnitine	2.54	2.27	<0.001
Asparaginyl-Valine	2.25	2.24	0.009
PA(20:5-3OH/10:0)	2.36	2.23	<0.001
Valylphenylalanine	2.59	2.21	0.010
N2-Acetylornithine	2.48	2.20	0.012
Leucyl-Glutamate	2.54	2.20	0.011
PI(16:0/18:0)	2.57	2.20	0.042
Deoxycholyltyrosine	2.20	2.19	0.012
LysoPC(18:1/0:0)	2.42	2.19	0.005
Docosapentaenoic acid	2.44	2.19	0.004
Norcholic acid	2.13	2.14	0.024
Heptadecanoyl carnitine	2.36	2.14	0.028
Threonylglycine	1.95	2.13	0.047
Deoxycholylglutamic acid	2.19	2.12	0.045
10,11-Dihydro-12R-hydroxy-leukotriene E4	2.28	2.10	0.008
Leucyl-Glycine	2.72	2.10	0.017
Histidylalanine	2.29	2.09	0.023
Lysylhydroxyproline	2.56	2.07	0.010

Ketomethylvaleric acid	2.54	2.04	0.042
Pyroglutamylleucine	2.38	2.02	0.047
Type III			
Stercobilin	4.58	4.24	0.001
Stercobilinogen	4.34	3.99	0.003
Methacholine	4.14	3.64	<0.001
PA(18:1-2OH/8:0)	3.92	3.54	0.003
Deoxycholic acid	4.12	3.44	0.007
PC(18:1-2OH/2:0)	3.65	3.23	0.000
Nicotinic acid	3.68	3.07	0.026
LysoPE(16:0/0:0)	3.68	2.98	0.022
Lithocholic acid	3.52	2.92	0.004
Hypoxanthine	3.58	2.91	0.003
MG(20:4/0:0/0:0)	3.39	2.83	0.039
Dodecanedioic acid	3.20	2.81	0.011
Nutriacholic acid	3.45	2.75	0.021
Glutaric acid	3.14	2.73	0.015
Inosine	2.86	2.54	0.001
MG(20:3/0:0/0:0)	1.96	2.53	0.005
N1-Acetylspermidine	3.12	2.50	0.004
Methyl sulfate	2.22	2.49	<0.001
Sphinganine	2.86	2.46	0.009
Thiamine	2.61	2.45	0.001
MG(14:1/0:0/0:0)	2.53	2.44	0.006
N-Arachidonoyl GABA	2.10	2.40	0.041
Decenoic acid	1.94	2.37	0.040
Adenosine	2.94	2.36	0.048

LysoPA(18:4/0:0)	2.97	2.36	0.040
Methylglutaric acid	2.63	2.35	0.009
Proline betaine	2.93	2.32	0.003
Malonylcarnitine	1.94	2.26	0.033
Allolithocholic acid	2.73	2.25	0.034
Arginylphenylalanine	2.25	2.25	0.043
Ursodeoxycholic acid	2.78	2.24	0.009
CPA(18:0/0:0)	2.01	2.24	0.024
PA(8:0/14:0)	2.10	2.22	0.001
PA(20:4-OH/i-22:0)	2.34	2.21	0.001
Glutamylglutamine	2.48	2.21	0.005
N-Acetyl-L-methionine	2.54	2.16	0.022
DG(22:6-2OH/0:0/20:0)	2.67	2.16	0.030
7-Oxostigmasterol	2.68	2.15	0.013
2-Indolecarboxylic acid	2.43	2.13	0.005
LysoPA(8:0/0:0)	2.57	2.10	0.017
DG(PGF1alpha/2:0/0:0)	2.52	2.09	0.008
6-Octenoylcarnitine	2.43	2.06	0.038
Stearoylglycerophosphoglycerol	2.42	2.06	0.030
MG(18:3/0:0/0:0)	2.45	2.04	0.048

Abbreviations: CRC, colorectal cancer; LDA, linear discriminant analysis.

[a] Colorectal neoplasms were classified into three distinct gut microbiota enterotypes (subtypes) - type I, type II, and type III - using the Dirichlet multinomial mixture model based on their gut microbiota compositions. The Logarithm Value column displays the logarithm of the highest mean value among the two subtypes for each metabolite, representing the magnitude of the metabolite's peak area in the subtype with the highest mean. The LDA Value column presents the logarithmic LDA score for each discriminative metabolite, quantifying the effect size or the degree of separation between subtypes for each metabolite, with higher LDA scores indicating more significant differences between subtypes. The P Value column shows the p-value from the statistical test (e.g., Kruskal-Wallis test) used to assess the significance of the differences in metabolite peak area between subtypes, where smaller p-values indicate a higher level of statistical significance.

Table S10. Differential abundance of metabolites between type I and type III subtypes in CRA group [a]

Metabolite_Subtype	Logarithm value	LDA value	P value
Type I			
Urobilinogen	4.81	4.18	0.002
L-Phenylalanine	4.43	3.87	0.045
L-Valine	4.25	3.57	0.005
Allocholic acid	3.90	3.49	<0.001
N,N,N-Trimethyl-L-alanyl-L-proline betaine	3.89	3.46	0.004
Linoleoyl ethanolamide	3.88	3.29	0.010
Chenodeoxycholic acid sulfate	3.80	3.24	0.038
Ursodeoxycholic acid 3-sulfate	3.63	3.21	0.001
Cholic acid	3.89	3.19	0.018
Leucylproline	3.58	3.13	0.008
2-Piperidinone	3.64	3.09	0.004
Isodeoxycholic acid	3.57	3.02	0.001
3-Oxocholic acid	3.49	2.99	0.001
Tyramine	3.40	2.87	0.000
Eicosapentaenoyl Ethanolamide	3.44	2.86	0.012
7-Ketodeoxycholic acid	3.10	2.72	<0.001
3-Hydroxyhexanoic acid	3.10	2.72	0.036
Mesobilirubinogen	3.24	2.70	0.028
Urothion	2.10	2.69	0.013
Oleoylethanolamide	3.25	2.68	0.015
Docosa-pentaenoyl carnitine	1.79	2.67	0.002
N1,N8-Diacetylspermidine	3.25	2.62	0.024
N-Acetylhistamine	3.06	2.61	0.028
L-Palmitoylcarnitine	3.10	2.59	0.002
Cholylasparagine	1.98	2.57	<0.001

3-Methylthymine	3.11	2.55	0.018
LysoPE(18:4/0:0)	2.96	2.54	0.025
Tryptamine	2.86	2.54	<0.001
LysoPA(18:3/0:0)	2.92	2.52	0.003
12-Hydroxy-12-octadecanoylcarnitine	1.87	2.49	0.020
Oleoylcarnitine	3.11	2.49	0.003
PC(5-iso PGF2VI/2:0)	2.18	2.47	0.036
Muricholic acid	3.03	2.46	0.021
2-Pentenoic acid	3.13	2.45	0.026
2-Hydroxyvaleric acid	2.78	2.44	0.006
5-Methyltetrahydrofolic acid	1.97	2.43	0.006
DG(20:4-OH/0:0/2:0)	3.10	2.41	0.030
Myristoleoylcarnitine	2.95	2.40	0.005
MG(16:0/0:0/0:0)	2.73	2.40	0.010
PGP(16:1/16:0)	2.43	2.35	0.006
27-Norcholestanehexol	2.91	2.34	0.005
Gluconolactone	2.27	2.33	0.025
Cholylhistidine	2.32	2.33	0.007
PA(LTE4/8:0)	1.94	2.32	0.019
D-Ribulose	2.56	2.32	0.004
Prolylproline	2.60	2.32	0.044
L-2-Hydroxyglutaric acid	2.62	2.32	0.003
Alpha-Linolenoyl ethanolamide	2.81	2.30	0.017
7-Sulfocholic acid	2.74	2.30	0.005
LysoPC(16:0/0:0)	2.89	2.29	0.044
Leukotriene E3	1.96	2.28	0.029
Linolenic acid	2.42	2.28	0.001
Deoxycholylproline	2.55	2.28	<0.001

LysoPI(18:1/0:0)	2.10	2.27	0.002
DG(20:4-2OH/0:0/2:0)	2.88	2.27	0.026
Pentadecanoylcarnitine	2.65	2.26	0.001
Docosapentaenoic acid	2.46	2.26	<0.001
Tyrosyl-Leucine	2.64	2.25	0.016
LysoPA(22:6/0:0)	2.78	2.25	0.004
3-Hydroxyoctadecenoylcarnitine	2.35	2.24	<0.001
MG(24:6/0:0/0:0)	2.52	2.24	0.006
PA(20:5-3OH/10:0)	2.49	2.23	<0.001
Niacinamide	2.53	2.22	0.044
Tetradecanoylcarnitine	2.52	2.22	0.002
Leucyl-Glycine	2.75	2.21	0.033
LysoPA(i-14:0/0:0)	2.63	2.19	0.006
Histidylleucine	2.42	2.19	0.045
Prolylhydroxyproline	2.62	2.18	0.006
9,10,13-TriHOME	2.68	2.17	0.011
Taurodehydrocholic acid	2.25	2.17	0.012
3'-Deoxythymidine	2.53	2.16	0.015
Pentadecanone	2.35	2.16	0.050
20-Hydroxy-leukotriene E4	2.31	2.15	0.047
N2-Acetylornithine	2.45	2.15	0.016
Deoxycholyltyrosine	2.38	2.15	0.012
Deoxycholylglutamic acid	2.49	2.14	0.022
hydroxyoctadecadienoic acid	2.48	2.14	0.047
linolenyl carnitine	2.39	2.13	0.026
10,11-Dihydro-12R-hydroxy-leukotriene E4	2.58	2.11	0.020
N-Stearoyl Serine	2.48	2.08	0.013
Norcholic acid	2.38	2.00	0.026

Type III			
Stercobilin	4.41	3.93	<0.001
Stercobilinogen	4.26	3.73	0.002
PA(18:1-2OH/8:0)	3.87	3.19	0.030
Deoxycholic acid	4.04	3.12	0.023
Dinor-12-oxophytodienoic Acid	2.04	2.70	0.006
4-Hydroxyestrone sulfate	1.96	2.65	0.001
Glutaric acid	2.99	2.61	<0.001
Xanthosine	2.11	2.61	0.010
LysoPE(P-16:0/0:0)	2.96	2.58	0.001
Inosine	2.88	2.54	0.001
3-Hydroxyhexadecanoic acid	2.93	2.49	<0.001
PA(8:0/14:0)	1.99	2.49	0.010
Uridine	2.05	2.41	0.033
MG(PGF2alpha/0:0/0:0)	3.22	2.36	0.029
7,8-Dihydropteroic acid	2.36	2.33	0.005
N-Arachidonoyl GABA	1.93	2.32	0.020
Proline betaine	2.83	2.31	0.045
PG(18:0/TXB2)	2.26	2.28	0.020
PA(20:4-OH/i-22:0)	2.16	2.26	0.045
N-Acetyl-L-glutamic acid	2.94	2.25	0.035
LysoPA(8:0/0:0)	2.71	2.25	<0.001
Sphinganine	2.67	2.25	0.040
Methylglutaric acid	2.26	2.23	0.015
Cholylglutamic acid	2.36	2.22	0.003
Allolithocholic acid	2.50	2.20	0.011
DG(22:6-2OH/0:0/20:0)	2.48	2.15	0.015
Dimethyl glutarate	2.32	2.12	0.008

LysoPA(22:1/0:0)	2.42	2.11	0.018
MG(PGE1/0:0/0:0)	2.51	2.09	0.023
3b-Hydroxy-5-cholenoic acid	2.29	2.08	0.034

Abbreviations: CRA, colorectal adenoma; LDA, linear discriminant analysis.

[a] Colorectal neoplasms were classified into three distinct gut microbiota enterotypes (subtypes) - type I, type II, and type III - using the Dirichlet multinomial mixture model based on their gut microbiota compositions. The Logarithm Value column displays the logarithm of the highest mean value among the two subtypes for each metabolite, representing the magnitude of the metabolite's peak area in the subtype with the highest mean. The LDA Value column presents the logarithmic LDA score for each discriminative metabolite, quantifying the effect size or the degree of separation between subtypes for each metabolite, with higher LDA scores indicating more significant differences between subtypes. The P Value column shows the p-value from the statistical test (e.g., Kruskal-Wallis test) used to assess the significance of the differences in metabolite peak area between subtypes, where smaller p-values indicate a higher level of statistical significance.

Table S11. Metabolites with significant differential abundance in both CRC and CRA group comparisons between type I and type III subtypes [a]

Metabolite_Subtype	Colorectal cancer			Colorectal adenoma		
	Logarithm value	LDA value	P value	Logarithm value	LDA value	P value
Type I						
L-Valine	4.29	3.72	0.031	4.25	3.57	0.005
Chenodeoxycholic acid sulfate	3.95	3.60	0.006	3.80	3.24	0.038
Cholic acid	3.78	3.35	0.006	3.89	3.19	0.018
Allocholic acid	3.65	3.30	0.013	3.90	3.49	< 0.001
Ursodeoxycholic acid 3-sulfate	3.61	3.22	0.004	3.63	3.21	0.001
N,N,N-Trimethyl-L-alanyl-L-proline betaine	3.54	3.09	0.025	3.89	3.46	0.004
3-Oxocholic acid	3.31	2.95	0.004	3.49	2.99	0.001
Oleoylcarnitine	3.22	2.87	< 0.001	3.11	2.49	0.003
L-Palmitoylcarnitine	3.20	2.86	< 0.001	3.10	2.59	0.002
LysoPC(16:0/0:0)	3.24	2.84	0.022	2.89	2.29	0.044
Leucylproline	3.19	2.78	0.004	3.58	3.13	0.008

Oleoylethanolamide	3.24	2.76	0.039	3.25	2.68	0.015
7-Ketodeoxycholic acid	3.02	2.72	0.025	3.10	2.72	< 0.001
2-Pentenoic acid	3.14	2.62	0.036	3.13	2.45	0.026
LysoPA(18:3/0:0)	2.77	2.47	0.008	2.92	2.52	0.003
Tryptamine	2.79	2.46	0.003	2.86	2.54	< 0.001
Docosa-pentaenoyl carnitine	1.70	2.43	0.048	1.79	2.67	0.002
7-Sulfocholic acid	2.85	2.42	0.028	2.74	2.30	0.005
Muricholic acid	2.81	2.35	0.011	3.03	2.46	0.021
Histidylleucine	2.39	2.33	0.003	2.42	2.19	0.045
Cholylasparagine	1.92	2.33	0.014	1.98	2.57	< 0.001
Tetradecanoylcarnitine	2.58	2.33	< 0.001	2.52	2.22	0.002
Deoxycholylproline	2.67	2.32	< 0.001	2.55	2.28	< 0.001
3-Hydroxyoctadecenoylcarnitine	2.58	2.30	< 0.001	2.35	2.24	< 0.001
LysoPA(22:6/0:0)	2.67	2.27	0.002	2.78	2.25	0.004
LysoPE(18:4/0:0)	2.50	2.27	0.001	2.96	2.54	0.025
Pentadecanoylcarnitine	2.54	2.27	< 0.001	2.65	2.26	0.001
PA(20:5-3OH/10:0)	2.36	2.23	< 0.001	2.49	2.23	< 0.001
N2-Acetylornithine	2.48	2.20	0.012	2.45	2.15	0.016
Deoxycholyltyrosine	2.20	2.19	0.012	2.38	2.15	0.012
Docosapentaenoic acid	2.44	2.19	0.004	2.46	2.26	< 0.001
Norcholic acid	2.13	2.14	0.024	2.38	2.00	0.026
Deoxycholylglutamic acid	2.19	2.12	0.045	2.49	2.14	0.022
10,11-Dihydro-12R-hydroxy-leukotriene E4	2.28	2.10	0.008	2.58	2.11	0.020
Leucyl-Glycine	2.72	2.10	0.017	2.75	2.21	0.033
Type III						
Stercobilin	4.58	4.24	0.001	4.41	3.93	< 0.001

Stercobilinogen	4.34	3.99	0.003	4.26	3.73	0.002
PA(18:1-2OH/8:0)	3.92	3.54	0.003	3.87	3.19	0.030
Deoxycholic acid	4.12	3.44	0.007	4.04	3.12	0.023
Glutaric acid	3.14	2.73	0.015	2.99	2.61	< 0.001
Inosine	2.86	2.54	0.001	2.88	2.54	0.001
Sphinganine	2.86	2.46	0.009	2.67	2.25	0.040
N-Arachidonoyl GABA	2.10	2.40	0.041	1.93	2.32	0.020
Methylglutaric acid	2.63	2.35	0.009	2.26	2.23	0.015
Proline betaine	2.93	2.32	0.003	2.83	2.31	0.045
Allolithocholic acid	2.73	2.25	0.034	2.50	2.20	0.011
PA(8:0/14:0)	2.10	2.22	0.001	1.99	2.49	0.010
PA(20:4-OH/i-22:0)	2.34	2.21	0.001	2.16	2.26	0.045
DG(22:6-2OH/0:0/20:0)	2.67	2.16	0.030	2.48	2.15	0.015
LysoPA(8:0/0:0)	2.57	2.10	0.017	2.71	2.25	< 0.001

Abbreviations: CRA, colorectal adenoma; CRC, colorectal cancer; LDA, linear discriminant analysis.

[a] Colorectal neoplasms were classified into three distinct gut microbiota enterotypes (subtypes) - type I, type II, and type III - using the Dirichlet multinomial mixture model based on their gut microbiota compositions. The Logarithm Value column displays the logarithm of the highest mean value among the two subtypes for each metabolite, representing the magnitude of the metabolite's peak area in the subtype with the highest mean. The LDA Value column presents the logarithmic LDA score for each discriminative metabolite, quantifying the effect size or the degree of separation between subtypes for each metabolite, with higher LDA scores indicating more significant differences between subtypes. The P Value column shows the p-value from the statistical test (e.g., Kruskal-Wallis test) used to assess the significance of the differences in metabolite peak area between subtypes, where smaller p-values indicate a higher level of statistical significance.

Table S12. Pathway analysis results for differential metabolites between type I and type III subtypes in colorectal cancer group [a]

Pathway	Total	Hits	Metabolite	Subtype	Raw p	-log10(p)	Holm p	FDR	Impact
Fatty acid degradation	39	1	L-Palmitoylcarnitine	type I	<0.001	5.23	<0.001	<0.001	0
Tryptophan metabolism	41	1	Tryptamine	type I	0.003	2.51	0.025	0.007	0.040
Primary bile acid biosynthesis	46	1	Cholic acid	type I	0.018	1.76	0.105	0.030	0
Glycerophospholipid metabolism	36	1	LysoPC(16:0/0:0)	type I	0.071	1.15	0.357	0.075	0.017
Valine, leucine and isoleucine degradation	40	1	L-Valine	type I	0.075	1.12	0.357	0.075	0
Valine, leucine and isoleucine biosynthesis	8	1	L-Valine	type I	0.075	1.12	0.357	0.075	0
Pantothenate and CoA biosynthesis	19	1	L-Valine	type I	0.075	1.12	0.357	0.075	0
Aminoacyl-tRNA biosynthesis	48	1	L-Valine	type I	0.075	1.12	0.357	0.075	0
Purine metabolism	65	3	Adenosine; Hypoxanthine; Inosine	type III	0.000	3.44	0.004	0.002	0.020
Thiamine metabolism	7	1	Thiamine	type III	0.001	3.25	0.006	0.002	0
Sphingolipid metabolism	21	1	Sphinganine	type III	0.002	2.62	0.022	0.007	0.154
Nicotinate and nicotinamide metabolism	15	1	Nicotinic acid	type III	0.011	1.97	0.075	0.021	0

Abbreviations: FDR, false discovery rate.

[a] Colorectal neoplasms were classified into three distinct gut microbiota enterotypes (subtypes) - type I, type II, and type III - using the Dirichlet multinomial mixture model based on their gut microbiota compositions. The Total column shows the total number of compounds in the pathway; the Hits column presents the number of compounds that match with the differential metabolites; the Metabolite column lists the matched differential metabolites; the Subtype column indicates the subtype(s) where the corresponding pathway is significantly enriched; the Raw p column shows the original p value calculated from the enrichment analysis; the Holm p column shows the p value adjusted by the Holm-Bonferroni method; the FDR column shows the p value adjusted using False Discovery Rate; and the Impact column shows the pathway impact value calculated from pathway topology analysis.

Table S13. Pathway analysis results for differential metabolites between type I and type III subtypes in colorectal adenoma group[a]

Pathway	Total	Hits	Metabolite	Subtype	Raw p	-log10(p)	Holm p	FDR	Impact
Tryptophan metabolism	41	1	Tryptamine	type I	<0.001	5.05	<0.001	<0.001	0.040
Tyrosine metabolism	42	1	Tyramine	type I	<0.001	3.40	0.008	0.003	0.025
Fatty acid degradation	39	1	L-Palmitoylcarnitine	type I	0.002	2.62	0.043	0.012	0
One carbon pool by folate	9	1	5-Methyltetrahydrofolic acid	type I	0.004	2.38	0.071	0.012	0
Primary bile acid biosynthesis	46	1	Cholic acid	type I	0.004	2.36	0.071	0.012	0
alpha-Linolenic acid metabolism	13	1	Linolenic acid	type I	0.004	2.35	0.071	0.012	0.333
Biosynthesis of unsaturated fatty acids	36	1	Linolenic acid	type I	0.004	2.35	0.071	0.012	0
Pantothenate and CoA biosynthesis	19	1	L-Valine	type I	0.010	2.01	0.117	0.017	0
Valine, leucine and isoleucine biosynthesis	8	1	L-Valine	type I	0.010	2.01	0.117	0.017	0
Valine, leucine and isoleucine degradation	40	1	L-Valine	type I	0.010	2.01	0.117	0.017	0
Aminoacyl-tRNA biosynthesis	48	2	L-Phenylalanine; L-Valine	type I	0.013	1.90	0.117	0.020	0
Glycerophospholipid metabolism	36	1	LysoPC(16:0/0:0)	type I	0.029	1.54	0.201	0.040	0.017
Phenylalanine, tyrosine and tryptophan biosynthesis	4	1	L-Phenylalanine	type I	0.055	1.26	0.276	0.064	0.500
Phenylalanine metabolism	10	1	L-Phenylalanine	type I	0.055	1.26	0.276	0.064	0.357
Pentose phosphate pathway	22	1	Gluconolactone	type I	0.059	1.23	0.276	0.066	0
Purine metabolism	65	2	Xanthosine; Inosine	type III	<0.001	3.86	0.003	0.001	0.002
Folate biosynthesis	27	1	7,8-Dihydropteroic acid	type III	0.007	2.17	0.087	0.016	0
Nicotinate and nicotinamide metabolism	15	1	Nicotinic acid	type III	0.013	1.88	0.117	0.020	0.194
Pyrimidine metabolism	39	1	Uridine	type III	0.034	1.46	0.207	0.045	0.016
Arginine biosynthesis	14	1	N-Acetyl-L-glutamic acid	type III	0.071	1.15	0.276	0.075	0
Sphingolipid metabolism	21	1	Sphinganine	type III	0.085	1.07	0.276	0.085	0.154

Abbreviations: FDR, false discovery rate.

[a] Colorectal neoplasms were classified into three distinct gut microbiota enterotypes (subtypes) - type I, type II, and type III - using the Dirichlet multinomial

mixture model based on their gut microbiota compositions. The Total column shows the total number of compounds in the pathway; the Hits column presents the number of compounds that match with the differential metabolites; the Metabolite column lists the matched differential metabolites; the Subtype column indicates the subtype(s) where the corresponding pathway is significantly enriched; the Raw p column shows the original p value calculated from the enrichment analysis; the Holm p column shows the p value adjusted by the Holm-Bonferroni method; the FDR column shows the p value adjusted using False Discovery Rate; and the Impact column shows the pathway impact value calculated from pathway topology analysis.

Table S14. Correlation between differential metabolites and differential bacterial genera in type I CRC subgroup [a]

Metabolite	Actinomyces			Alistipes			Bacillus			Bacteroides			Eggerthella			Family XIII AD3011 group			Lachnospiraceae. incertae sedis			Odoribacter		
	CC	p	FDR	CC	p	FDR	CC	p	FDR	CC	p	FDR	CC	p	FDR	CC	p	FDR	CC	p	FDR	CC	p	FDR
Allolithocholic acid	-0.17	0.16	0.64	0.45	<0.01	0.05	-0.57	<0.01	0.01	0.19	0.14	0.60	-0.05	0.71	0.93	0.14	0.28	0.75	0.01	0.96	0.99	0.13	0.29	0.76
Asparaginyl-Valine	0.06	0.62	0.90	-0.13	0.28	0.75	-0.05	0.71	0.93	0.16	0.20	0.69	0.46	<0.01	0.05	-0.17	0.17	0.65	-0.14	0.26	0.73	-0.05	0.71	0.93
Deoxycholylproline	0.15	0.23	0.72	-0.04	0.78	0.95	0.09	0.46	0.84	0.08	0.55	0.88	0.07	0.56	0.89	-0.09	0.47	0.85	-0.51	<0.01	0.02	0.07	0.60	0.90
DG(22:6-2OH/0:0/20:0)	-0.16	0.20	0.68	0.44	<0.01	0.06	-0.54	<0.01	0.01	0.18	0.14	0.61	-0.05	0.67	0.92	0.10	0.43	0.82	-0.02	0.89	0.98	0.10	0.43	0.83
Dodecanedioic acid	-0.31	0.01	0.25	0.27	0.03	0.35	-0.55	<0.01	0.01	0.37	<0.01	0.13	0.06	0.64	0.91	0.09	0.45	0.84	0.02	0.87	0.97	-0.03	0.80	0.95
Glutaric acid	-0.18	0.14	0.62	0.34	0.01	0.19	-0.47	<0.01	0.05	0.32	0.01	0.24	0.01	0.92	0.98	0.06	0.63	0.91	0.01	0.91	0.98	0.02	0.85	0.97
Inosine	-0.03	0.81	0.96	0.21	0.09	0.53	-0.48	<0.01	0.04	0.22	0.08	0.51	0.12	0.34	0.78	-0.14	0.26	0.73	-0.03	0.84	0.96	0.09	0.48	0.85
Lithocholic acid	-0.23	0.06	0.47	0.42	<0.01	0.07	-0.46	<0.01	0.05	0.29	0.02	0.31	-0.01	0.96	0.99	0.08	0.52	0.87	-0.06	0.64	0.91	0.18	0.15	0.62
L-Valine	0.46	<0.01	0.05	-0.30	0.01	0.28	0.36	<0.01	0.16	-0.21	0.09	0.53	0.09	0.49	0.86	-0.34	0.01	0.19	0.19	0.12	0.58	-0.25	0.04	0.41
Methylglutaric acid	-0.18	0.15	0.62	0.50	<0.01	0.02	-0.51	<0.01	0.02	0.29	0.02	0.31	0.03	0.83	0.96	0.00	0.98	0.99	0.01	0.96	0.99	0.11	0.38	0.80
N,N,N-Trimethyl-L-alanyl-L-proline betaine	0.34	0.01	0.20	-0.32	0.01	0.24	0.38	<0.01	0.13	-0.13	0.30	0.76	0.05	0.68	0.93	-0.47	<0.01	0.04	-0.10	0.41	0.82	-0.30	0.01	0.28
N1-Acetylspermidine	0.16	0.21	0.70	-0.22	0.08	0.50	0.04	0.75	0.94	0.07	0.58	0.89	0.30	0.02	0.28	-0.50	<0.01	0.02	-0.02	0.90	0.98	-0.36	<0.01	0.17
PA(20:4-OH/i-22:0)	-0.19	0.12	0.58	0.37	<0.01	0.15	-0.46	<0.01	0.05	0.27	0.03	0.34	0.06	0.63	0.91	0.12	0.33	0.78	-0.03	0.82	0.96	0.10	0.43	0.83
PA(20:5-3OH/10:0)	0.19	0.13	0.59	-0.28	0.02	0.32	0.00	1.00	1.00	0.10	0.43	0.83	0.19	0.12	0.58	-0.27	0.03	0.35	-0.13	0.32	0.77	-0.48	<0.01	0.03
Tryptamine	0.04	0.77	0.95	-0.03	0.81	0.96	0.01	0.92	0.98	0.48	<0.01	0.03	0.25	0.04	0.40	-0.25	0.04	0.41	-0.21	0.10	0.55	-0.13	0.31	0.77

Abbreviations: CC, correlation coefficient; CRC, colorectal cancer; FDR, false discovery rate.

[a] Colorectal neoplasms were classified into three distinct gut microbiota enterotypes (subtypes) - type I, type II, and type III - using the Dirichlet multinomial mixture model based on their gut microbiota compositions. Only differential metabolites and differential bacterial genera that have at least one pair of correlation analysis with an FDR-corrected p-value less than 0.05 are displayed in the table. The CC column represents the strength and direction of the relationship between differential metabolites and differential bacterial genera. The p column from the correlation analysis indicates the probability of observing a correlation as strong as the one calculated by chance. The FDR column adjusts the original p-value to control the false discovery rate. Cells with FDR values less than 0.05 are highlighted in yellow to indicate statistical significance.

Supplementary Figure

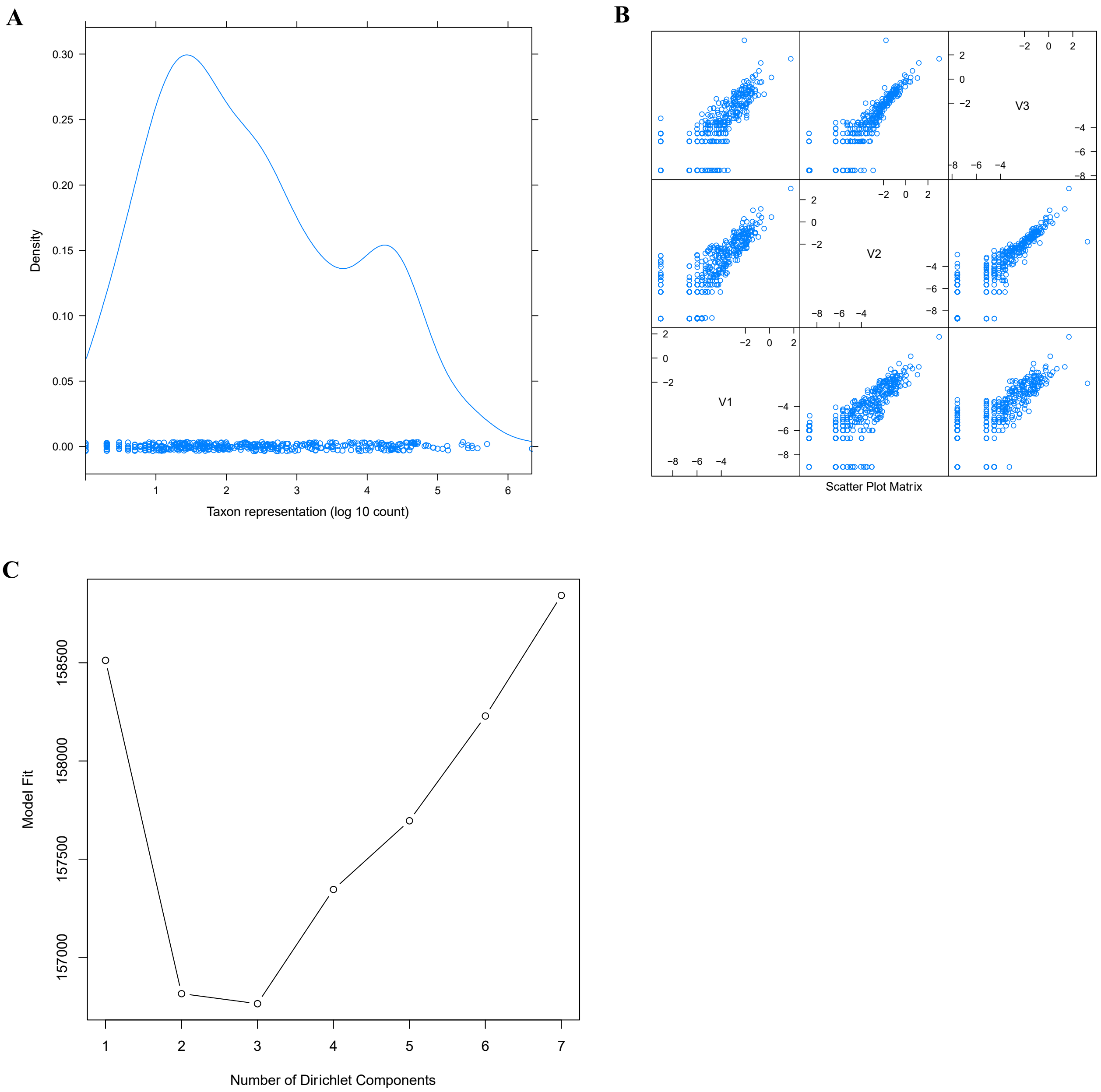

Figure S1. Combined visualization of taxon representation, scatterplot matrix, and Laplace approximate log-likelihood values for the Dirichlet Multinomial mixture model. (A) Density plot and scatter plot of log10(count) taxon representation for 466 bacterial genera across 250 cases, illustrating the overall distribution and individual log10(count) values. The smooth curve denotes the probability density distribution of the logarithmically-transformed abundance of bacterial genera, while the individual points beneath the curve correspond to the log10(count) values for each of the 466 bacterial genera. (B) Scatterplot matrix of log-transformed fitted values for the optimal Dirichlet Multinomial mixture model. Each panel exhibits the relationship between log-transformed fitted values of two components (V1, V2, and V3) within the mixture model. A positive correlation can be observed between the components in each panel. (C) Laplace approximate log-likelihood values

plotted against the number of Dirichlet components for the Dirichlet Multinomial mixture model. The x-axis represents the number of Dirichlet components, ranging from 1 to 7, while the y-axis displays the corresponding Laplace approximate log-likelihood values. The plot suggests that the optimal number of clusters for the best-fitting model is 3, as it coincides with the minimum Laplace approximate log-likelihood value.

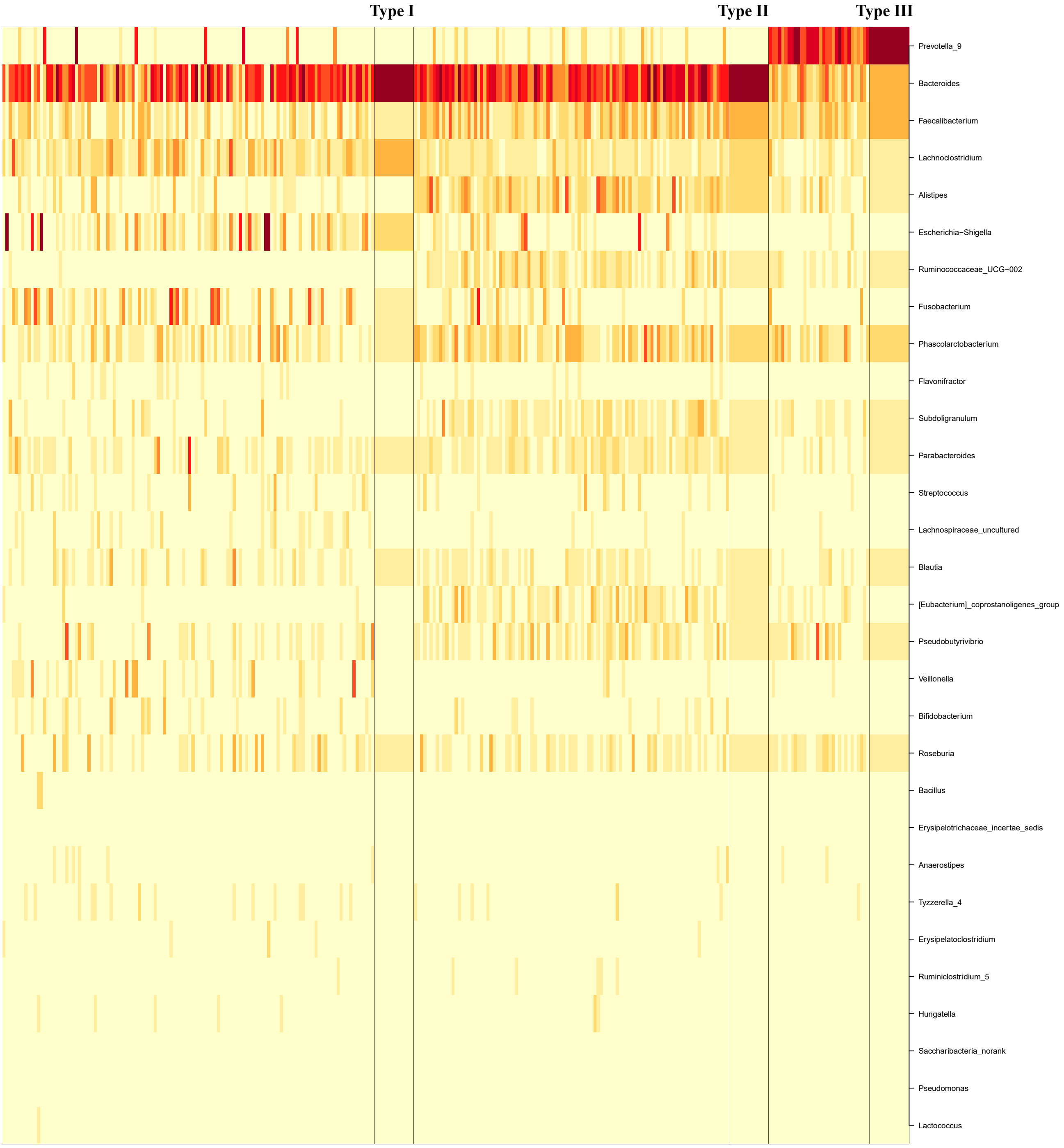

Figure S2. Heatmap of the top 30 taxa with the largest differences between the baseline model and the best Dirichlet multinomial mixture model. Each row represents one of the 30 taxa, sorted in descending order. The narrow columns illustrate individual samples, while the wide columns indicate the three enterotypes (type I, type II, and type III). On the left side, multiple narrow columns correspond to all samples of type I, followed by a wide column for type I. This pattern repeats for type II and type III. The color intensity of red signifies the degree of difference, where darker shades represent larger differences.

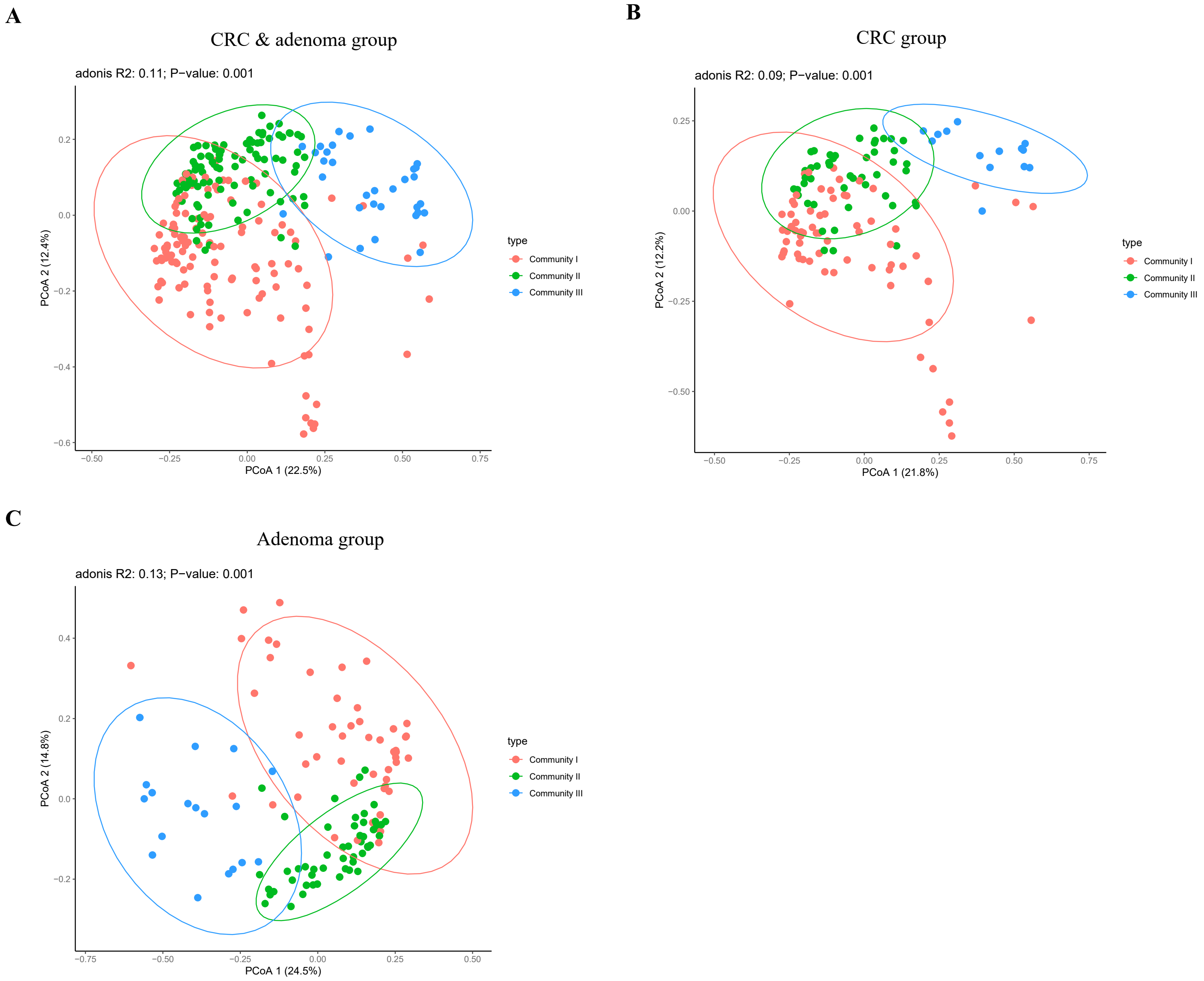

Figure S3. Principal coordinate analysis (PCoA) plots based on Bray-Curtis distances depict the three distinct microbial community groups (type

I, type II, and type III) in (A) colorectal neoplasm, (B) colorectal cancer (CRC), and (C) colorectal adenoma (CRA) samples. Each plot displays PCoA 1 on the x-axis and PCoA 2 on the y-axis. In all plots, samples are represented by red (type I), green (type II), and blue (type III) points, with surrounding ellipses illustrating the 95% confidence interval for each group. The relationships and overlaps among the ellipses are detailed for each plot. These visualizations emphasize the associations and intersections between the three identified microbial community profiles in colorectal neoplasm, CRC, and CRA samples.

A **B**

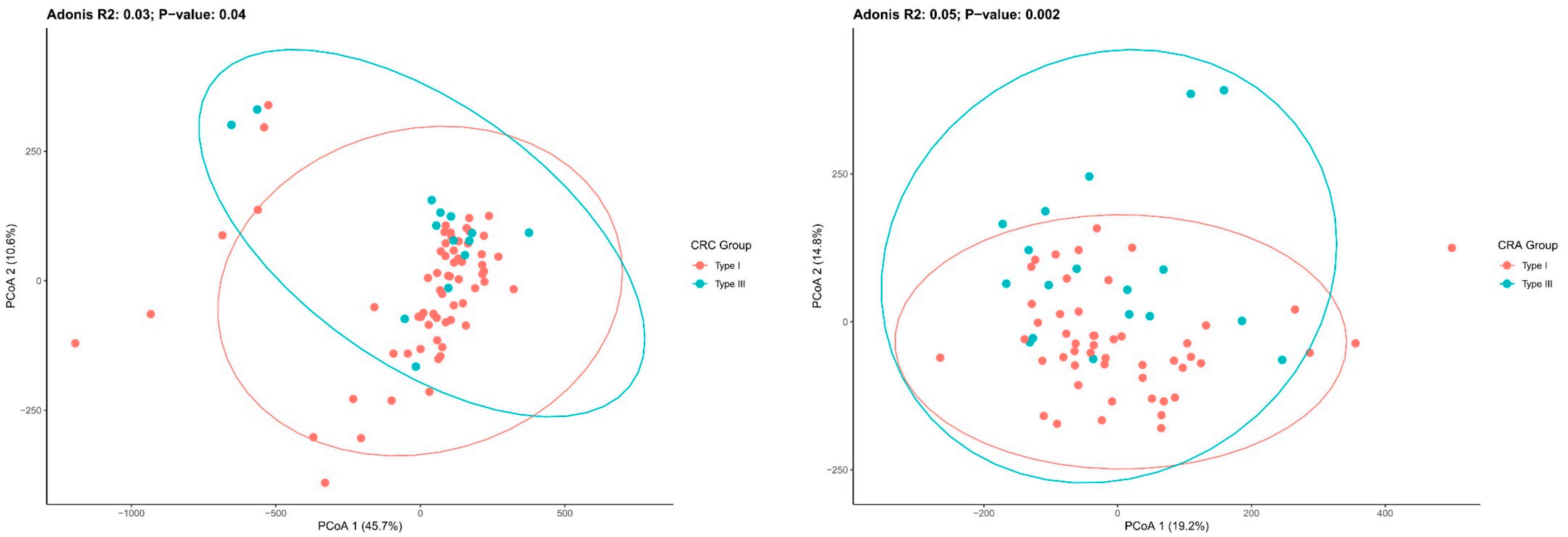

Figure S4. Principal coordinate analysis (PCoA) plots based on Manhattan distances illustrate the distinct metabolic profiles between subtypes (type I and type III) in (A) colorectal cancer (CRC) and (B) colorectal adenoma (CRA) samples. Each plot displays PCoA 1 on the x-axis and PCoA 2 on the y-axis. Samples are represented by red (type I) and blue (type III) points, with surrounding ellipses illustrating the 95% confidence interval for each group. The relationships and overlaps among the ellipses are detailed for each plot.

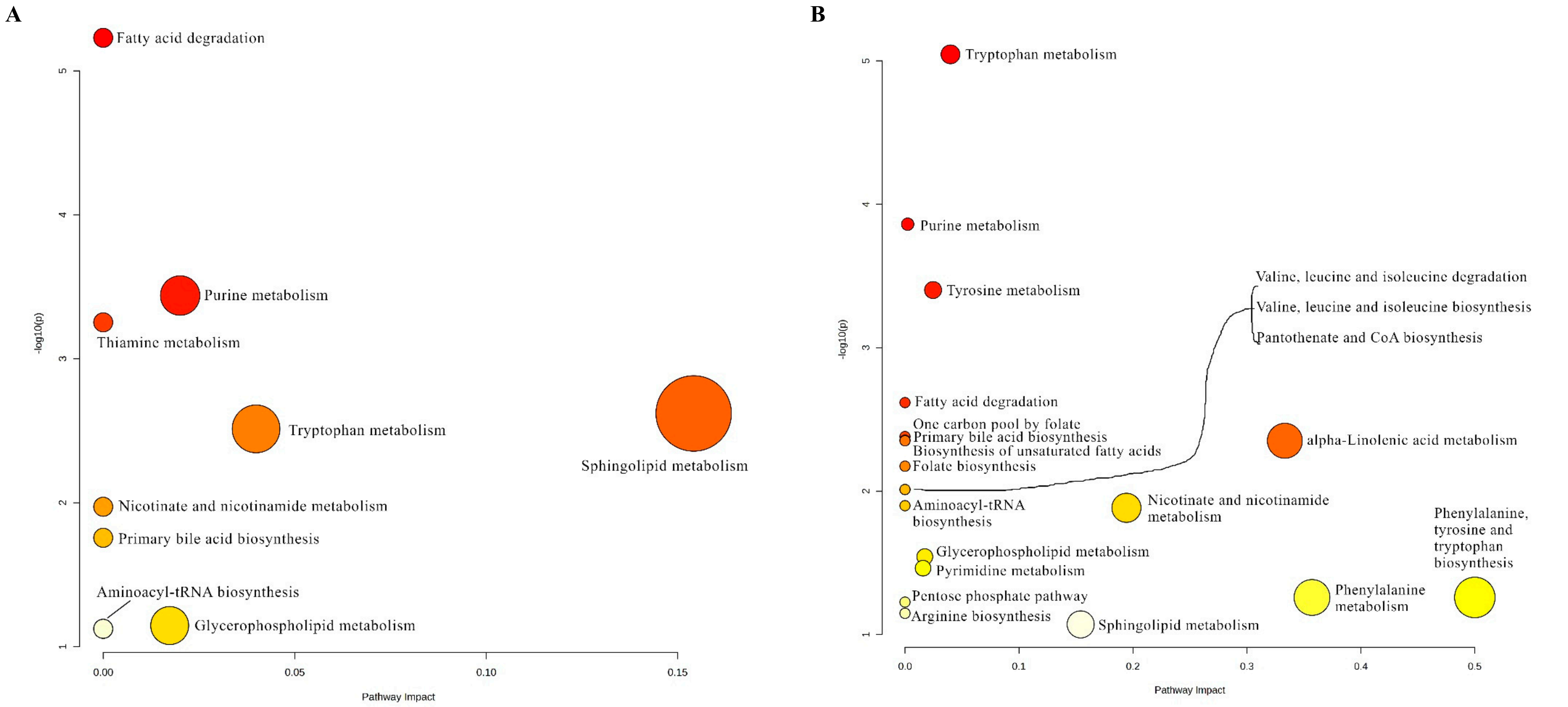

Figure S5. Overview of pathway analysis for differential metabolites between type I and type III subtypes in (A) colorectal cancer and (B) colorectal adenoma groups. All matched pathways are depicted as circles according to the original p values obtained from the pathway enrichment analysis and the pathway impact values from the pathway topology analysis, where larger circles represent higher pathway impact values. The circles are colored from red to yellow, with darker shades indicating smaller original p values and higher -log10(p) values from the enrichment analysis.
